# *Pseudomonas aeruginosa* strains belonging to phylogroup 3 frequently exhibit an atypical quorum sensing response: the case of MAZ105, a tomato rhizosphere isolate

**DOI:** 10.1099/mic.0.001401

**Published:** 2023-10-11

**Authors:** Sara E. Quiroz-Morales, Luis Felipe Muriel-Millán, Gabriel Y. Ponce-Soto, Abigail González-Valdez, Israel Castillo-Juárez, Luis Servín-González, Gloria Soberón-Chávez

**Affiliations:** ^1^​ Departamento de Biología Molecular y Biotecnología, Instituto de Investigaciones Biomédicas, Universidad Nacional Autónoma de México, Ciudad Universitaria, Apdo, Postal 70228, C. P. 04510, CDMX, Mexico; ^2^​ Departamento de Microbiología Molecular, Instituto de Biotecnología, Universidad Nacional Autónoma de México, Av. Universidad 2001, Col. Chamilpa, Cuernavaca, Morelos CP 62210, Mexico; ^3^​ Microbial Paleogenomics Unit, Department of Genomes and Genetics, Pasteur Institute, 75015 Paris, France; ^4^​ Laboratorio de Investigación y Aplicación de Fitoquímicos Bioactivos, Colegio de Postgraduados, 56230, Campus Montecillo, Texcoco, Mexico

**Keywords:** anti-virulence therapy, Pqs quorum sensing, *Pseudomonas aeruginosa* group 3, pyocyanin

## Abstract

*
Pseudomonas aeruginosa* is a widespread γ-proteobacterium and an important opportunistic pathogen. The genetically diverse *P. aeruginosa* phylogroup 3 strains are characterized by producing the pore-forming ExlA toxin and by their lack of a type III secretion system. However, like all strains of this species, they produce several virulence-associated traits, such as elastase, rhamnolipids and pyocyanin, which are regulated by quorum sensing (QS). The *P. aeruginosa* QS response comprises three systems (Las, Rhl and Pqs, respectively) that hierarchically regulate these virulence factors. The Pqs QS system is composed of the PqsR transcriptional factor, which, coupled with the alkyl-quinolones HHQ or PQS, activates the transcription of the *pqsABCDE* operon. The products of the first four genes of this operon produce HHQ, which is then converted to PQS by PqsH, while PqsE forms a complex with RhlR and stabilizes it. In this study we report that mutations affecting the Pqs system are particularly common in phylogroup 3 strains. To better understand QS in phylogroup 3 strains we studied strain MAZ105 isolated from tomato rhizosphere and showed that it contains mutations in the central QS transcriptional regulator, LasR, and in the gene encoding the PqsA enzyme involved in the synthesis of PQS. However, it can still produce QS-regulated virulence factors and is virulent in *Galleria mellonella* and mildly pathogenic in the mouse abscess/necrosis model; our results show that this may be due to the expression of *pqsE* from a different PqsR-independent promoter than the *pqsA* promoter. Our results indicate that using anti-virulence therapy based on targeting the PQS system will not be effective against infections by *P. aeruginosa* phylogroup 3 strains.

## Graphical abstract

A *Pseudomonas aeruginosa* MAZ105 strain belonging to phylogroup 3 has a defective Pqs QS system due to a polar mutation in *pqsA* but can produce pyocyanin because it can express PqsE in low-phosphate medium.
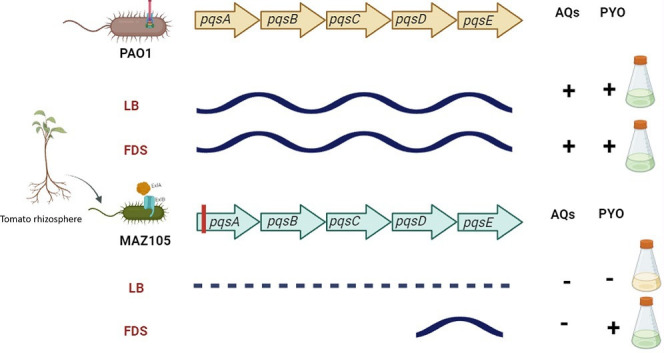

## Introduction

*Pseudomonas aeruginosa* is an important opportunistic pathogen defined by the World Health Organization in 2017 as a critical priority [1] due to its production of virulence factors [2, 3] and its high intrinsic and acquired antibiotic resistance [4]. However, it is also a widespread environmental bacterium that has even been claimed to be ubiquitous [5, 6].

This bacterial species is constituted by five phylogroups [7], four of which have a high degree of genomic conservation (clades 1, 2, 4 and 5), while the genetically diverse group 3 (PA7 group) has been defined as an outlier clade due to its high genetic sequence variability [7–9]. This group has recently been proposed to constitute a separate bacterial species [10]. However, strains belonging to any of the five *P. aeruginosa* phylogroups are potentially pathogenic even if isolated from environmental samples and show high conservation of strategies and traits involved in establishing pathogenic interactions [7, 9, 11].

Among the conserved virulence-associated traits in all *P. aeruginosa* phylogroups are those regulated at the transcriptional level by quorum sensing (QS), such as the LasB elastase (ELA), the rhamnolipid biosurfactant (RL) and the redox-active phenazine pyocyanin (PYO) that has antibiotic activity [12]. QS is a global regulatory circuit [13] that is constituted by three hierarchically arranged systems [12, 14]. The Las, Rhl and Pqs systems consist of the transcriptional regulators LasR, RhlR and PqsR, respectively, which activate transcription when coupled with their cognate autoinducers (AIs). LasR binds 3-oxo-dodecanoyl-homoserine lactone (3O-C12-HSL) autoinducer (AI) produced by LasI and activates the transcription of several genes encoding virulence factors, such as ELA (*lasB*), as well as *rhlR*, *lasI*, *rhlI*, *pqsH* and *pqsR*, and is thus at the top of the regulatory cascade. In turn, RhlR coupled with the AI butanoyl-homoserine lactone (C4-HSL) produced by RhlI activates transcription of genes responsible for RL and PYO production, among others. The third QS regulatory system (Pqs) depends on the 2-alkyl-4-quinolones (AQs), 2-heptyl-3,4-dihydroxyquinoline (PQS) and its precursor 4-hydroxy-2-heptylquinoline (HHQ), which interact with PqsR (14). PqsR coupled with PQS or HHQ AIs modulates QS by activating *pqsABCDE* operon transcription. This operon encodes the enzymes responsible for HHQ synthesis, and the enzyme PqsH catalyses the conversion of HHQ to PQS. The role of the PQS system in *P. aeruginosa* QS response is mainly due to the ability of PqsE to stabilize and modulate the transcriptional activity of RhlR, presumably by forming a protein complex [14–16]. This is especially apparent in PYO production, since no PYO is produced in a *pqsE* mutant, even though HHQ and PQS are synthesized [14].

Natural *lasR* mutants are frequently isolated in both clinical and environmental settings. In the natural *lasR* mutants that have been characterized recently, the QS-dependent expression of virulence factors is regulated by RhlR [17–20]. Furthermore, we have recently reported that when the *P. aeruginosa* PAO1 type strain is cultured in phosphate-limited conditions, LasR becomes dispensable for the QS-dependent production of virulence factors, such as PYO and RL, and RhlR becomes the head of the QS network [21].

However, in the low-phosphate-stress condition, the regulation of the PAO1 Pqs QS system is not modified, and expression of the *pqsABCDE* operon remains dependent on a functional PqsR transcriptional regulator. Therefore, a Δ*pqsR* PAO1 mutant cannot produce AQs and PYO even in a phosphate-limited condition [22]. This Δ*pqsR* PAO1 mutant phenotype is due to the lack of *pqsE* expression since its production of PYO can be restored by PqsR and by PqsE [22]. In contrast, in the phylogroup 3 strain ATCC 9027, which carries a frameshift mutation in *pqsR* [22], PYO is produced under low-phosphate conditions, even though there is no transcription starting from the PqsR-dependent *pqsA* promoter and no production of AQs is attained [22]. PYO production in this strain seems to be due to the expression of *pqsE* from a PqsR-independent promoter that has not been localized [21]. The phylogroup 3 type strain PA7 also contains a frameshift mutation in *pqsR* [8], which is different from that of ATCC 9027 and can produce PYO under low-phosphate conditions [21]. However, the molecular mechanism involved in the production of this phenazine has not been determined in this type strain.

In a recent study, the frequency of mutations affecting QS genes was determined in the genomes of 852 *P *. *aeruginosa* strains, including some environmental but mostly clinical isolates from different type of infections [19]. This study showed that loss of function *lasR* mutations were very common among these strains independently of their source of isolation, while only a few *pqsR* mutants were detected, and that the amino acid sequence of some QS proteins such as PqsA, was highly variable. However, strains from phylogroup 3 were not specifically analysed in this work [19]. In our experience, mutations affecting *pqsR* were frequent among *P. aeruginosa* strains belonging to phylogroup 3, such as strains ATCC 9027 [22] and PA7 [8, 22].

The present work aims to determine whether the QS system of *P. aeruginosa* strains belonging to phylogroup 3 showed a higher frequency of loss-of-function *pqsR* mutations, and in such a case, to obtain some insights into the molecular mechanism involved in the expression of the virulence factors that are dependent on the Pqs QS system in strains belonging to the PA7 group.

To this end we analysed the QS-related genes in the 47 genomes of phylogroup 3 strains deposited in the Pseudomonas Genome Database (Table S1, available in the online version of this article). This analysis shows that, besides a high frequency of *lasR* mutations, phylogroup 3 strains with inactivating mutations in *pqsR* are indeed more frequent than previously reported [19]. Interestingly, strain MAZ105, which was isolated from the rhizosphere of a tomato plant collected in the Mexican state of Guerrero in April 2017, has a wild-type *pqsR* gene, but presents mutations in both the *lasR* and *pqsA* genes. We further analysed its QS response, mainly the molecular mechanism involved in PYO production, and showed that it lacks AQs production due to the *pqsA* mutation. However, it can produce RL, low levels of ELA, and PYO, and is virulent in *Galleria mellonella* and mildly virulent in mice abscess models. In addition, we present evidence suggesting that in this environmental isolate PYO production occurs independently of a functional PqsR, because in low-phosphate conditions PqsE is expressed independently of the *pqsABCDE* operon, as has been shown in the phylogroup 3 strain ATCC 9027 [22].

The results presented in this work show that *P. aeruginosa* phylogroup 3 strains commonly have an atypical QS response. Furthermore, we show that it is common that in QS-defective phylogroup 3 strains, AQ production is dispensable because PqsE can be expressed independently from the *pqsABCDE* operon or a functional PqsR in a low-phosphate medium [22]. Therefore, the possibility of using anti-QS therapeutic strategies to treat infections caused by phylogroup 3 strains will be limited if they are focused on the Pqs-dependent QS response.

## Experimental procedures

### Microbiological conditions

The strains and plasmids used in this work are shown in Table S2. Precultures were grown in LB broth [23]. Antibiotics were used at the following concentrations: for *Escherichia coli *, 200 µg ml^−1^ carbenicillin (Cb), 15 µg ml^−1^ tetracycline (Tc), 30 µg ml^−1^ streptomycin (Sm); for *P. aeruginosa,* 200 µg ml^−1^ Cb, 120 µg ml^−1^ Tc, 200 µg ml^−1^ Sm. FDS medium (20 ml l^−1^ glycerol, 10 g l^−1^ dl-alanine, 50 µM iron (III) citrate, 0.1M Na_2_SO_4_, 20 mM MgCl_2_, 0.5 mM K_2_HPO_4_, pH adjusted to 7.4) as previously reported [20] and LB medium was used to analyse phenotypic traits and for RNA extractions. Cultures were inoculated with washed cells at an optical density (600 nm) of 0.05. The flasks were incubated with continuous shaking (225 r.p.m.) at 37 °C. Antibiotics were only added to LB medium overnight cultures.

### Determination of RL, PYO, ELA and AQ production

Phenotypic traits were analysed at 24 h of growth using described previously methods. RLs were detected by thin-layer chromatography (TLC) [24], which was performed as follows. Each culture (5 ml) was centrifuged at 14 000 r.p.m. for 10 min, and the cell-free supernatant was acidified to pH 2 with concentrated HCl. The RL were extracted with an equal volume of chloroform–methanol (2 : 1). The organic phase of the two extractions was collected and the solvent was evaporated to dryness. The crude extract was dissolved in 50 µl methanol and 5 µl of the extract and 5 µl of each standard were separated by TLC on silica plates (silica gel 60; Merk) using a mixture of chloroform, methanol and acetic acid (65 : 15 : 2). RL were visualized by spraying with an alpha-naphthol solution (Sigma-Aldrich) prepared in an ethanol–H_2_SO_4_ mixture, and heating at 90 °C for 5 min.

PYO determination is based on its absorbance at 520 nm in acidic solution [25]. Briefly, a 5 ml sample of culture supernatant was extracted with 3 ml of chloroform and then re-extracted with 1 ml of 0.2 N HCl. The absorbance of this solution was measured at 520 nm and PYO concentration, expressed as μg ml^−1^ of culture supernatant, was determined by multiplying the optical density at 520 nm by 17.072 (standard extinction coefficient) and dividing by the optical density (600 nm) of the culture to avoid an effect due to growth differences.

ELA activity was determined using elastin Congo red [26]. Briefly, samples of the filter-sterilized culture supernatants were diluted 1 : 10 with culture medium and 10 mg of elastin Congo red (Sigma) was added to 1 ml of the dilution in glass tubes. The mixture was incubated at 37 °C for 16 h with constant rotation (225 r.p.m.), the insoluble substrate was removed by centrifugation (1300 ***g*** for 10 min at 4 °C) and the absorbance of the supernatant was measured at 495 nm with a spectrophotometer using as blank elastin Congo red sample incubated with medium alone.

The concentration of the AQs PQS and HHQ was determined using a bioassay based on light emission of the PAO1 *pqsA::lux* strain when incubated in the presence of supernatants containing these AIs [27]. Briefly, an equal volume of acidified ethyl acetate was added to 5 ml of culture supernatant. After making two extractions, the ethyl acetate was evaporated and 50 µl of methanol was added. Finally, 25 µl of this mixture was quantified using the bioreporter PAO1 ∆*pqsA* CTX *pqsA::lux*, which is activated in the presence of PQS and HHQ in the medium [27].

All experiments were performed three times in triplicate with supernatants from three individual growth experiments. Statistical significance was determined with Prism 6 software from GraphPad. For multiple-comparison tests analysis of variance (ANOVA) was used and single comparisons were established using Student’s *t*-test.

### Plasmids and DNA manipulations

DNA was purified and manipulated using standard techniques [28]. All plasmids were routinely maintained in *E. coli* DH5α or TOP10 and were introduced by electrotransformation to *P. aeruginosa* as described previously [29]. The *pqsABCDE* operon was amplified by PCR using *P. aeruginosa* PAO1 genomic DNA as a template and the primers PqsAE_PAO1FW and PqsAE_PAO1RV (Table S3). This fragment was cloned into the *Bam*HI-*Hind*III sites of the pUCP20 plasmid, leading to pUCP20-pqsABCDE.

The genome sequence of MAZ105 was determined by LANGEBIO (Irapuato, Mexico) using Illumina technology with a MiSeq 2×300 sequencer using a pair end library and the TruSeq DNA Nano kit, with a genome coverage of 108×. The *de novo* assembly of the genome was done using Unicycler v0.4.8. This whole-genome shotgun project has been deposited at DDBJ/ENA/GenBank under the accession GCA_030131645.1.

### Mutagenesis strategies

The point mutation was generated on the pUCP20-pqsR plasmid using the oligonucleotides PqsRS201Fa and PqsRS201Fb (Table S3). The mutagenesis reaction was generated following the protocol of the Quikchange XL site-directed mutagenesis kit (Agilent Technologies). This reaction was introduced by transformation in *E. coli* DH5α, and the presence of the point mutation was verified by sequencing. The plasmid carrying the point mutation was used to transform electrocompetent cells of *P. aeruginosa* PAO1 Δ*pqsR* mutant.

### Reverse transcription PCR (RT-PCR) assays

Total RNA was extracted from cells grown in FDS and LB media for 24 h at 37 °C using Trizol reagent (Thermo Scientific). DNAse I (Thermo Scientific) was used to remove contaminant DNA in RNA samples. For the synthesis of the complementary DNA (cDNA), 200 ng of DNAse-treated RNA were used for the preparation of 20 µl-reactions using the RevertAid First Strand cDNA Synthesis kit (Thermo Scientific), and the reverse oligonucleotides for *pqsE*, *pqsA* and *rpsL* (Table S3). Approximately 2 µl of resultant cDNAs were used as the template to amplify fragments of the coding regions of *pqsE* (155 bp), *pqsA* (100 bp) and *rpsL* (100 bp) using *Taq* DNA polymerase (Thermo Scientific) in 20 µl reactions with the following conditions: 1 cycle of DNA denaturation at 95 °C for 5 min, 26 cycles of denaturation at 95 °C (30 s), annealing at 60 °C (30 s) and elongation at 72 °C (30 s), and 1 cycle of final extension for 1 min at 72 °C. The RT-PCR products were visualized by loading 5 µl of each reaction into wells of 1.2 % agarose gels. The primer pairs used were RTpqsEAPFW/RTpqsEAPRv for *pqsE*, and rt_rpsL-F/rt_rpsL-R for *rpsL* (Table S3). The *rpsL* gene was used as a control for the cDNA reactions.

### Assays to measure the virulence of *P. aeruginosa* strains

The pathogenicity of *P. aeruginosa* strains was determined by their ability to induce abscesses, necrosis and systemic dissemination in mice. The mouse abscess model was performed as previously described [30–32]. Male and female CD-1 mice were used in a similar proportion, from 9 to 12 weeks of age, from the General Vivarium of the Facultad de Estudios Superiores Iztacala, Universidad Nacional Autónoma de México. The assays were conducted in the Laboratory of Investigation and Application of Bioactive Phytochemicals of the Colegio de Postgraduados. At all times, the indications of NOM-062-ZOO-1999 for the handling and use of laboratory animals were complied with, as well as the Consejo de Bienestar Animal, Colegio de Postgraduados (COBIAN/007/22 protocol). The animals were housed under standard conditions and provided food and water *ad libitum*.

For the *G. mellonella* model, we used larvae weighing 150–200 mg inoculated with 1×10^2^ cells of the indicated strains. Larvae were monitored daily for survival for 2 days. Ten larvae were used for the experimental group, and sterile saline solution was used as a negative control.

### Bioinformatic analysis

The *Pseudomonas* Genome Database (www.pseudomonas.com; accessed 26 January 2023) was used to obtain the genome sequences used in this work except for MAZ105.

Global nucleotide alignments to identify mutations in *lasR*, *pqsR*, *rhlR*, or the *pqsABCDE* operon were carried out with Clustal Omega [33] at the EMBL-EBI web server.

**Fig. 1.**
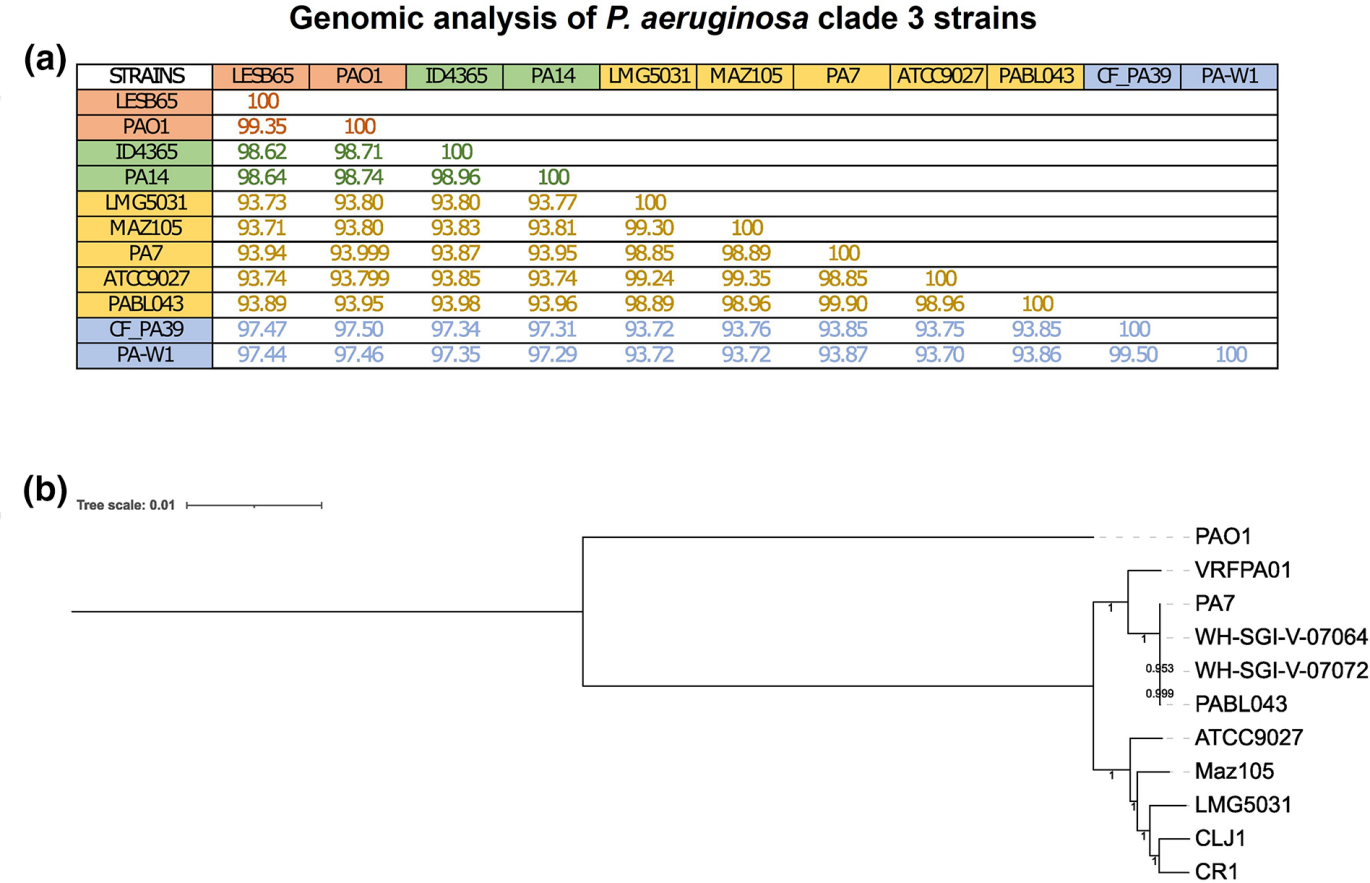
Genomic analysis of strains belonging to phylogroup 3. (**a**) The average nucleotide index (ANI) of strains belonging to phylogroup 3 (shown in yellow) phylogroups 1 (shown in orange), phylogroup 2 (presented in green) and phylogroup 5 (presented in blue) clearly shows that phylogroup 3 strains are genetically divergent. (**b**) The phylogenetic tree of the whole-genome sequence of some strains belonging to phylogoup 3 using the PAO1 genome as an outgroup shows that there are two subgroups among this clade.

The computation of the average nucleotide identity (ANI) was performed for 11 strains belonging to phylogroups 1, 2, 3 and 5 (Fig. 1). For phylogroup 1 we used PAO1 (GCF_000006765.1) and LESB65 (GCF_000583955.1). Strains representative of phylogroup 2 were UCPPB-PA14 (GCF_000014625.1) and ID4365 (GCA_000647615.1). In the case of phylogroup 3, we included PA7 (GCA_000017205.1), ATCC 9027 (GCA_001374975.1), LMG 5031 (GCA_003837245.1), PABLO043 (GCA_003411505.2) and MAZ105 (GCA_030131645.1). For phylogroup 5 we used PA-W1 (GCA_003833685.1) and CF PA39 (GCA_000568235.1). The ANI values were calculated using fastANI v1.33 [34]. To construct the phylogenetic tree, we used the genome sequences of *P. aeruginosa* members of phylogroup 3, using PAO1 genome as outgroup. The analysed strains correspond to PA7 (GCA_000017205.1), WH-SGI-V-07064 (GCA_001450465.1), PABLO043 (GCA_003411505.2), ATCC9027 (GCA_001374975.1), LMG 5031 (GCA_003837245.1), CLJ1 (GCA_003032395.1), CR1 (GCA_003025345.2), MAZ105 (GCA_030131645.1) and PAO1 (GCF_000006765.1). The genomes were aligned to identify locally collinear blocks (LCBs) using Mauve [35] with the progressive Mauve algorithm [36]. Subsequently, the LCBs shared by all genomes with a minimum length of 500 bases were extracted and concatenated, resulting in an alignment of 4 472 766 characters. Phylogenetic reconstruction was performed with FastTree [37] using the GTR model of nucleotide substitution and 1000 bootstraps.

## Results

### Mutations affecting the Pqs QS system and *lasR* are frequent among phylogroup 3 strains

To determine whether the presence of mutations in *pqsR* is a widespread characteristic of phylogroup 3 strains, we performed a multiple alignment of nucleotide sequences of this gene. We also aligned the predicted amino acid sequences of PqsR of the PAO1 type strain and the predicted PqsR sequences obtained from 47 genomes of phylogroup 3 strains that were deposited in the *Pseudomonas* Genome Database (Table S1). These included the 36 genomes that were previously characterized [11] and the MAZ105 genome, which was further analysed in this work. This multiple alignment showed that all phylogroup 3 strains contain phenylalanine in position 201, contrasting with the serine present in this position of PAO1 PqsR (Fig. S1). In addition, it was apparent that 16 strains contain mutations in *pqsR*, including 4 strains (PA7, PABLO43, WH-SGI-V-07064 and WH-SGI-V-07072) with the same frameshift mutation (Fig. S1) that changes the amino acid sequence of PqsR starting at position 232 (Table 1, Fig. S1).

**Table 1.** Putative loss of function mutations detected in *pqsR* among the 47 clade 3 strains analysed in this work

NAME	Source	Country	Nucleotide change	Amino acid change	Type of change	Assembly accession
WH-SGI-V-07370	Clinical isolation	France	553_555 insertion AAC	insertion N185	In-frame insertion	GCA_001450165.1
ATCC9027	Clinical isolation	Australia	755 insertion G	na	Frameshift mutation	GCA_001374975.1
PA7	Clinical isolation, non-respiratory	Argentina	698 and 699 deletion GA	na	Frameshift mutation	GCA_000017205.1
PABL043	Clinical isolation	USA	698 and 699 deletion GA	na	Frameshift mutation	GCA_003411505.2
WH-SGI-V-07064	Clinical isolation	USA	698 and 699 deletion GA	na	Frameshift mutation	GCA_001450465.1
WH-SGI-V-07072	Clinical isolation	USA	698 and 699 deletion GA	na	Frameshift mutation	GCA_001449385.1
ATCC33359	Environmental isolation	Denmark	437_442 deletion CCATCA	I147, T148 deletion	In-frame deletion	GCA_001444975.1
EML545	Clinical isolation	Germany	437_442 deletion CCATCA	I147, T148 deletion	In-frame deletion	GCA_001280745.1
AR_0356	Clinical isolation	Unknown	448_462 deletion GACGAGGAGTTGAAG	D150, E151, E152, L153, K154 deletion	In-frame deletion	GCA_002968755.1
AR441	Clinical isolation	Unknown	448_462 deletion GACGAGGAGTTGAAG	D150, E151, E152, L153, K155 deletion	In-frame deletion	GCA_003073695.1
WH-SGI-V-07165	Clinical isolation	France	448_462 deletion GACGAGGAGTTGAAG	D150, E151, E152, L153, K156 deletion	In-frame deletion	GCA_001449465.1
EML1796	Environmental isolation	France	448_462 deletion GACGAGGAGTTGAAG	D150, E151, E152, L153, K157 deletion	In-frame deletion	GCF_024464665.1
EML1795	Environmental isolation	France	448_462 deletion GACGAGGAGTTGAAG	D150, E151, E152, L153, K158 deletion	In-frame deletion	GCF_024464635.1
MIN-137	Clinical isolation	UK	702_714 deletion GGGCATCGCGCCG	na	Frameshift mutation	GCF_021460625.1
PSA00358	Clinical isolation	USA	C412T	Q138 stop	Stop gained	GCF_015732795.1
PSA00289	Clinical isolation	USA	C412T	Q138 stop	Stop gained	GCF_015734145.1

To determine the phylogenetic relationship with PA7 strain of the three phylogroup 3 strains that contain the same *pqsR* frameshift mutation as PA7, we constructed a phylogenetic tree using the whole-genome sequence of these strains and of seven other strains belonging to this phylogroup, using the PAO1 genome as an outgroup (Fig. 1). We found that the genomes of PA7 and the three strains that contain the same frameshift mutation (PABLO43, WH-SGI-V-0764 and WH-SGI-V-0772, Table 1) are very similar, despite being independent isolates (Table 1).

**Fig. 2.**
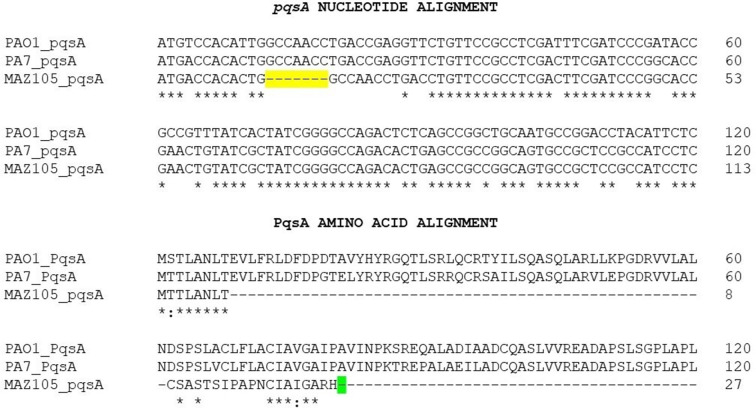
MAZ105 contains a mutated *pqsA* gene. Nucleotide and amino acid alignment of a fragment of MAZ105 *pqsA* and its encoded 27 amino acid protein, compared with the corresponding gene and protein of PAO1 and PA7 strains (these genes contain 1554 nt and encode a protein of 518 aa). The 7 nt deletion in MAZ105 *pqsA* is highlighted in yellow and the stop codon of the 27 aa protein encoded by this gene is shown in green.

To determine whether other phylogroup 3 strains with a defective PQS QS system were present among the 47 strains analysed (Table S1) we searched for mutations in the *pqsABCD* sequence in the 30 strains that do not have mutations in *pqsR*. We found three strains that contain mutations in *pqsA*: MAZ105 contains a 7 nt deletion (at positions 13–19) that causes a frameshift mutation (Fig. 2, Table 2), while LMG5031 and WH-SGI-V-07287 strains have a deletion of 3 nt at positions 382, 383 and 384, causing an in-frame deletion (Table 2). In addition, we found that the VRFPA01 strain contains a deletion of 1 nt at position 440 of *pqsB*, causing a frameshift mutation (Table 2). The strains carrying mutations in the *pqsABCDE* operon are unable to produce AQs and thus have an inactive PqsR transcriptional regulator. Therefore, our bioinformatic analysis shows that 20 out of the 47 clade 3 strains analysed have a defective Pqs QS system (Tables 1 and 2).

**Table 2.** Putative loss of function mutations detected in *pqsA* or *pqsB* among the 47 clade 3 strains analysed in this work

NAME	Source	Country/territory	Nucleotide change	Amino acid change	Type of change	Assembly accession
MAZ105	Environmental isolation	Mexico	Deletion of nucleotides 13–19 (GCCAACC) in *pqsA*	na	Frameshift mutation	GCA_030131645.1
LMG5031	Environmental isolation	Puerto Rico	Deletion of nucleotides 382–384 (CAG) in *pqsA*	Q128 deletion	In-frame deletion	GCA_003837245.1
WH-SGI-V-07287	Environmental isolation	Puerto Rico	Deletion of nucleotides 382–384 (CAG) in *pqsA*	Q128 deletion	In-frame deletion	GCA_001454675.1
VRFPAO1	Clinical isolation	India	Deletion of nucleotide 440 (C) in *pqsB*	na	Frameshift mutation	GCA_000335395.3

The high frequency of mutations affecting PqsR could be explained if this transcriptional regulator is non-functional in phylogroup 3 strains due to the S201F amino acid substitution compared to PAO1. To explore this possibility, we constructed a plasmid carrying a PAO1 *pqsR* gene with a mutation that caused this amino acid change at position 201 and compared PYO production of a PAO1 Δ*pqsR* mutant expressing this modified protein (PqsRS201F) or the unmodified PAO1 PqsR (Fig. S2). Our results show that PqsRF102S complemented the PAO1Δ*pqsR* mutant and even showed slightly higher PYO production than the same PAO1Δ*pqsR* mutant complemented with the unmodified *pqsR* gene (Fig. S2). These results show that the high frequency of mutations in *pqsR* in phylogroup 3 strains was not due to the inability of PqsRS201F present in these strains to activate transcription (Fig. S2).

We also analysed the sequence of the *lasR* gene in the 47 genomes of phylogroup 3 strains studied here to determine whether inactivating mutations were as frequent as in other phylogroups [19]. We found that 7 of the 47 phylogroup 3 strains contain mutations (Table 3), including strain MAZ105 that we characterize in this work.

**Table 3.** Putative loss of function mutations detected in *lasR* among the 47 clade 3 strains analysed in this work

NAME	Source	Country/territory	Nucleotide change	Amino acid change	Type of change	Assembly accession
WH-SGI-V-07064	Clinical isolation	USA	na	na	Deletion	GCA_001450465.1
MAZ105	Environmental isolation	Mexico	445 and 446 deletion CC	na	Frameshift mutation	GCA_030131645.1
AZPAE14941	Clinical isolation	PR China	392_403 deletion GCGTGGAGGCGG	S131, V132, E133, A134 deletion	In-frame deletion	GCA_000789725.1
515 477	na	USA	280_292 deletion CAGACACGCAAGC	na	Frameshift mutation	GCA_001909445.1
LMG5031	Environmental isolation	Puerto Rico	C292T	Q98STOP	Stop gained	GCA_003837245.1
EML528	Clinical isolation	Germany	G263A	W88STOP	Stop gained	GCA_001280755.1
WH-SGI-V-07287	Environmental isolation	Puerto Rico	C292T	Q98STOP	Stop gained	GCA_001454675.1

### The environmental isolate MAZ105 belongs to phylogroup 3 and has mutations in the QS genes *lasR* and *pqsA*

Strain MAZ105 was isolated from the rhizosphere of a tomato plant collected in the Mexican state of Guerrero in 2017. Its genomic characterization showed that it belongs to phylogroup 3 (Fig. 1), as can be seen from the ANI and its grouping in a phylogenetic tree with phylogroup 3 strains ATCC 9027, LMG5031, CLJ1 and CR1 (Fig. 1). In addition, we showed the presence of the *exlBA* operon in this strain (Fig. S3), which is characteristic of all isolates belonging to phylogroup 3 [38]. The MAZ105 genome also harbours the previously reported deletions affecting *rhlC*, *phzH* and the genes coding for the type III secretion system (T3SS) in strains of this clade [11].

The genomic analysis of strain MAZ105 showed that it does not contain mutations in *pqsR* (Table 1) but has a defective Pqs QS response as it presents a loss of function *pqsA* mutation (Fig. 2, Table 2). In addition, it contains an inactivating mutation in *lasR* (Table 3). The MAZ105 mutation in *pqsA* is a 7 nt deletion that spans from nucleotide 13 to nucleotide 19, causing a frameshift mutation that renders a 27 aa peptide and which is thus strongly polar on the *pqsABCDE* operon (Fig. 2).

**Fig. 3.**
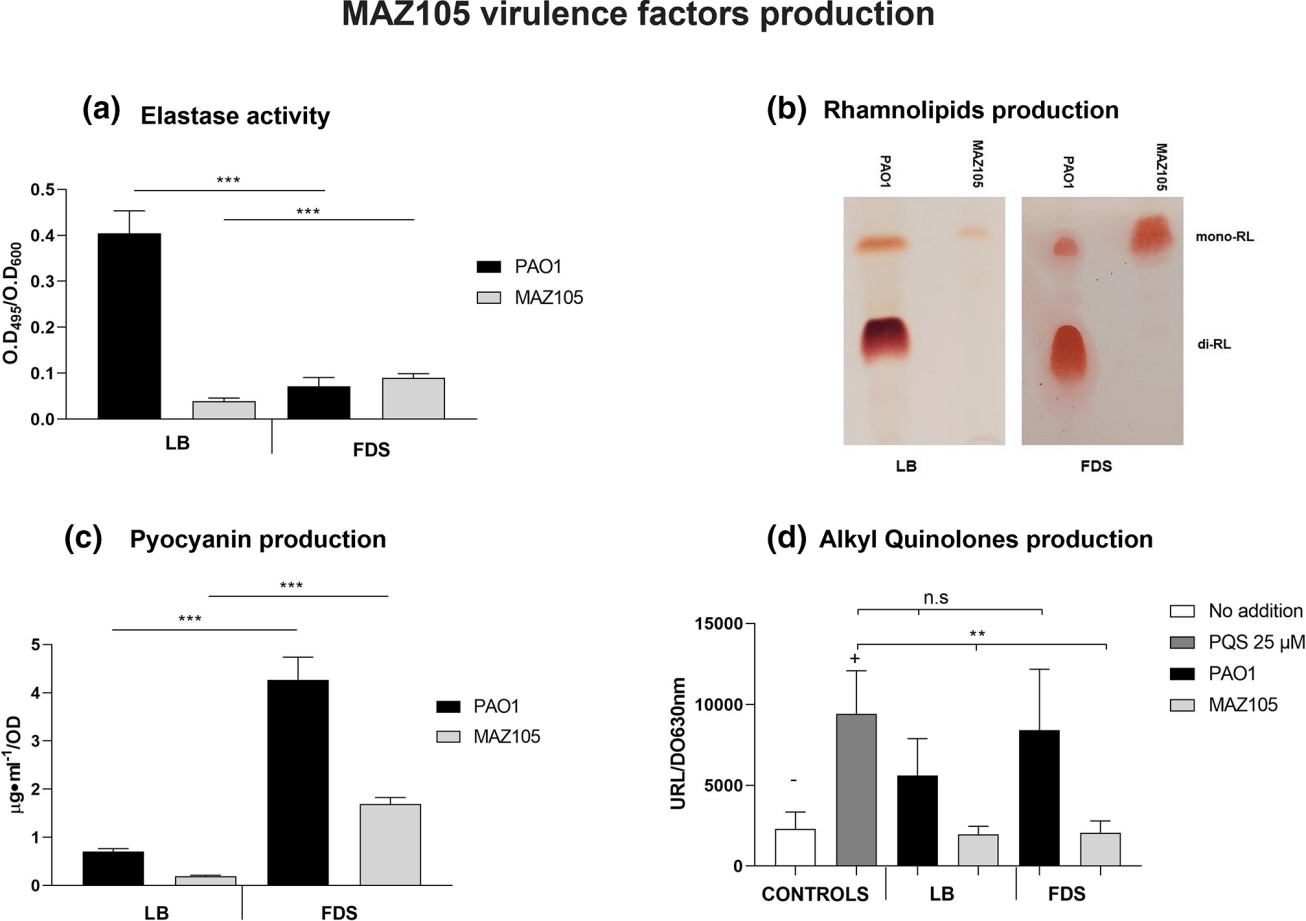
Production of virulence associated traits. (**a**) Production of ELA measured by the hydrolysis of elastine-congo red . (**b**) Production of RL detected by thin-layer chromatography. (**c**) Spectrophotometric measurement of PYO production. (**d**) Quantification of AQ production based on the luminescence produced by the AQ biosensor. The results shown are the means from three biological replicates, and error bars denote the standard deviations. The statistical analysis was established by Student’s *t*-test or in panel (d) by one-way ANOVA: *P*<0.001; Dunnett´s post-hoc test (***, *P*≤0.001; ns, not significant *P*>0.05). The symbols (−) and (+) indicate negative and positive controls, respectively. The four virulence-associated traits were measured in LB and FDS media after 16 h of growth. The production of these virulence-associated traits was measured in the PAO1 type strain under the same conditions to have a comparison with a strain that does not contain mutations in the QS genes.

Despite the mutations affecting QS genes, strain MAZ105 can produce ELA (at low levels), RL and PYO (Fig. 3a, b, c, respectively), although this last virulence-associated trait is only produced when this strain is cultured in the low-phosphate medium FDS (Fig. 3c). To have a comparison of the production of virulence associated traits with a wild-type strain that does not contain mutations affecting QS genes we used the PAO1 strain (Fig. 3).

### MAZ105 PYO production depends on *pqsE* expression and in the low-phosphate FDS is independent of the *pqsABCDE* operon and AQ production

**Fig. 4.**
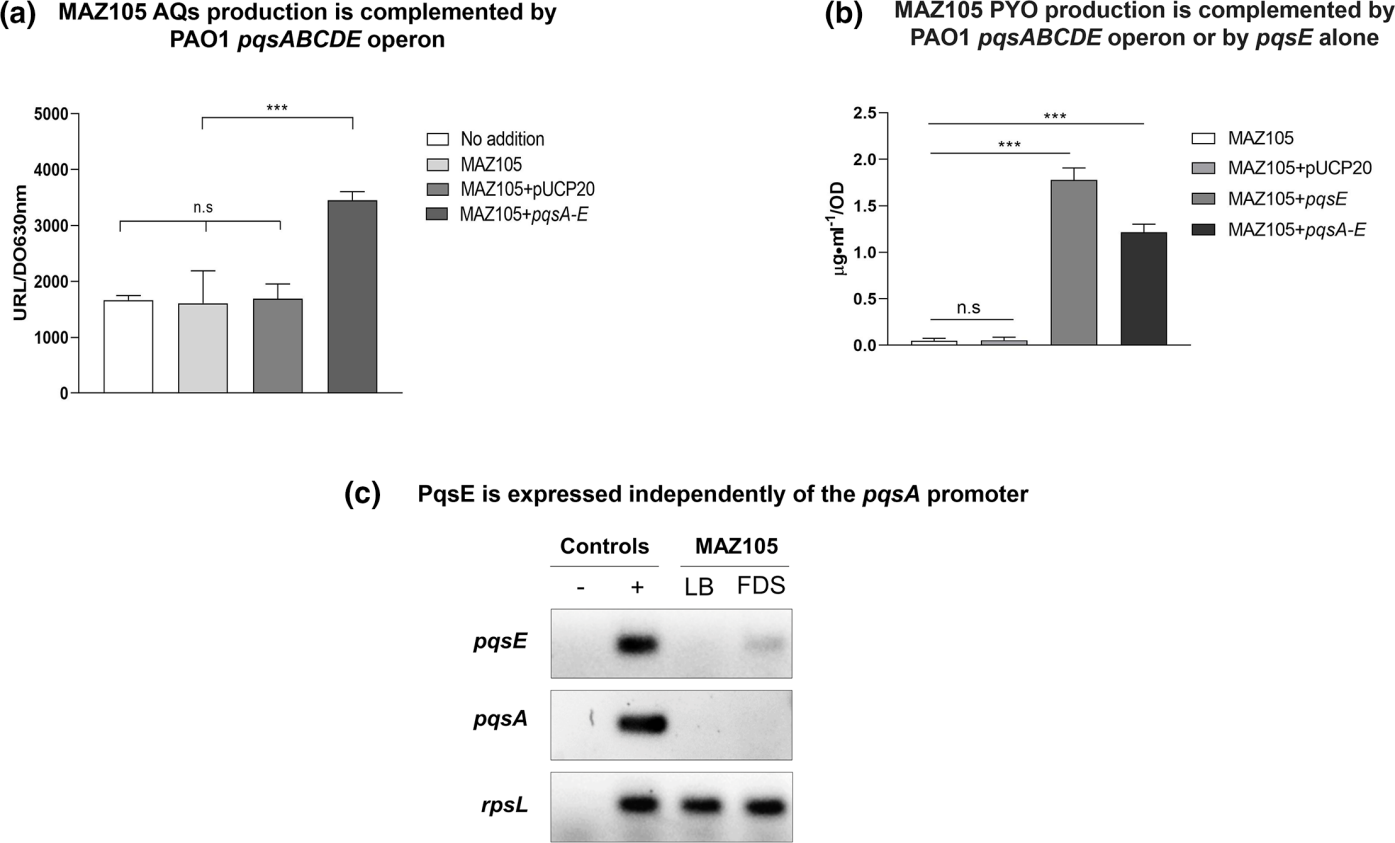
MAZ105 PYO production depends on *pqsE*, which is expressed independently of the *pqsABCDE* operon and PqsR. (**a**) Production of AQs in LB medium grown for 24 h. The statistical analysis was established by one-way ANOVA: *P*<0.001; Dunnett´s post-hoc test: ***P≤0.001; ns, not significant, *P*>0.05. (**b**) PYO production in LB medium after 24 h. of growth. The results shown are the means from three biological replicates and error bars denote the standard deviations. (**c**) Visualization of RT-PCR products of *pqsA, pqsE* and *rpsL* by electrophoresis in 1.2 % agarose gels. This result is representative of two independent experiments. Positive (+) and negative (−) controls refer to PCR reactions with and without genomic PAO1 DNA as a template, respectively.

PYO production is highly dependent on the Pqs system, so we focused on the characterization of the MAZ105 production of this phenazine and its relationship with the Pqs QS system. We hypothesized that the lack of MAZ105 PYO production in LB medium (Fig. 3c) was due to the lack of expression of the *pqsABCDE* operon as this strain does not produce AQs (Fig. 3d), presumably due to the *pqsA* mutation. To determine whether the polar effect of the MAZ105 *pqsA* mutation on the expression of the *pqsABCDE* operon was responsible for its lack of PYO and AQ production in LB medium (Figs 3 and 4), we cloned the PAO1 *pqsABCDE* operon in the pUCP20 plasmid (pUCP20-pqsABCDE), so it was constitutively expressed from the *lac* promoter. This plasmid restored the production of both PYO and of AQs in LB medium. (Fig. 4a, b). Furthermore, expression of the PAO1-derived *pqsE* (pUCP20-pqsE plasmid) [22] restored PYO production in LB medium (Fig. 4b), showing that it is the activity of PqsE but not AQ production that is the limiting factor for MAZ105 PYO production in LB medium. Therefore, the lack of PYO production in LB medium by MAZ105 is due to the lack of *pqsE* expression from the *pqsA* promoter in this condition.

To determine whether MAZ105 PYO production in FDS medium (Fig. 3c) was due to the expression of *pqsE*, independently of the *pqsABCDE* operon expression, we measured AQ production (Fig. 3d) and the expression of *pqsE* and *pqsA* by RT-PCR in LB and FDS medium (Fig. 4c). We found that in FDS medium MAZ105 produced PYO (Fig. 3c) and expressed *pqsE* despite its lack of AQs synthesis and of the expression from the *pqsA* promoter (Fig. 4c).

### Strain MAZ105 is virulent in the *G. mellonella* model and mildly virulent in the abscess formation assay in mice

**Fig. 5.**
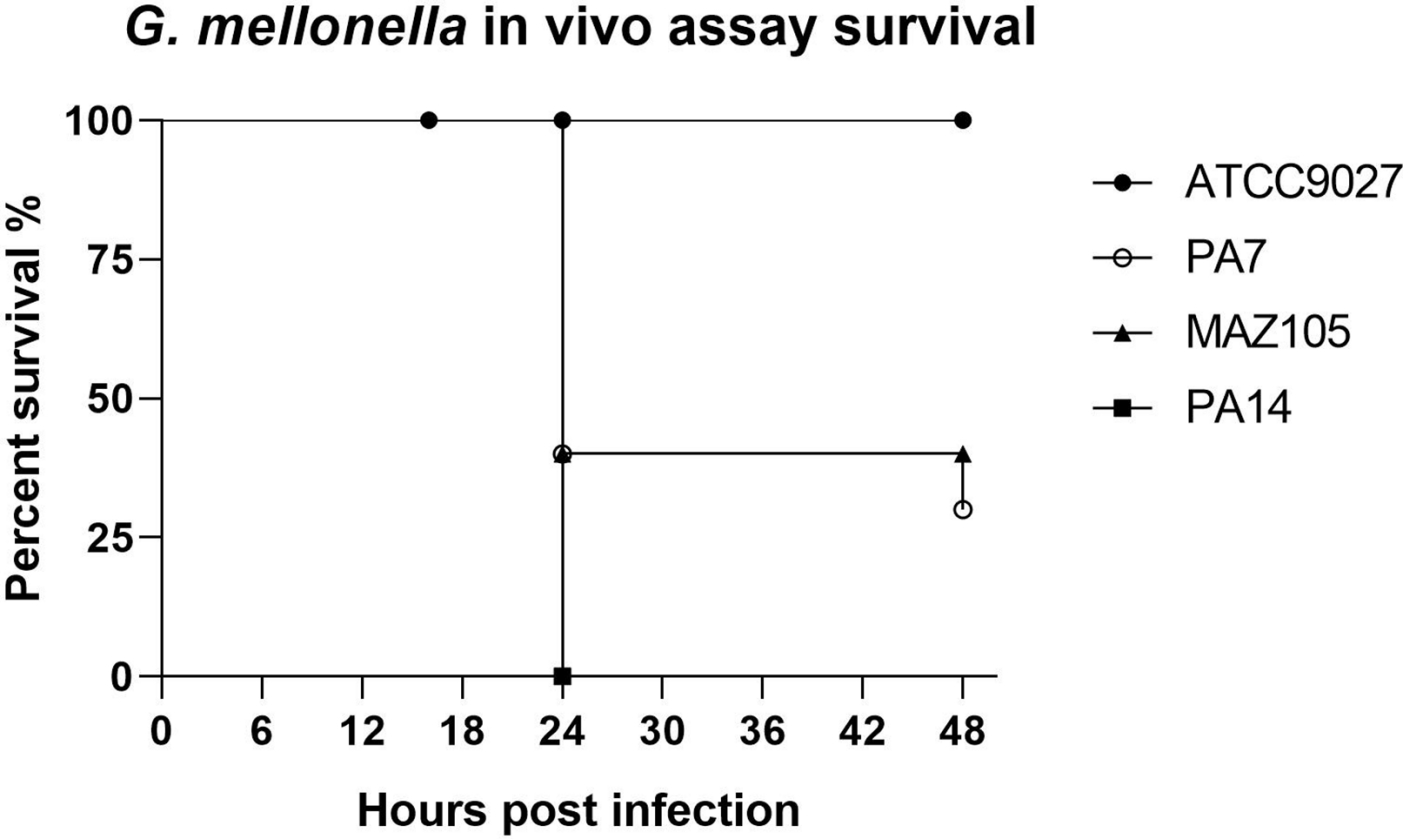
Virulence assays using the infection of *G. mellonella* larvae. Larvae weighing between 150 and 200 mg were injected with 1×10^2^ cells of the indicated strains and were monitored for survival at the time shown post-injection. Black circles, ATCC 9027 strain (negative control); white circles, PA7 (the phylogroup 3 type strain); black triangles, MAZ105; black squares, PAO1 (positive control).

To determine whether MAZ105 is virulent as has been reported for other *P. aeruginosa* environmental isolates [39], we evaluated its virulence using the PA7 type strain of phylogroup 3, which is a clinical isolate [8], as a positive control, and the ATCC 9027 strain, which is avirulent and also belongs to phylogroup 3 [40], as a negative control. To this end, we used two experimental virulence models: the formation of abscesses in mice (Table 4) [30, 32] and the injection of *G. mellonella* larvae (Fig. 5) [41]. Our results show that strain MAZ105 is only mildly virulent in mice, as is PA7 (Table 4), but is virulent in the *G. mellonella* model (Fig. 5). These results are consistent with previous reports showing that strains belonging to phylogroup 3 exhibit lower virulence compared to strains of phylogroups 1 and 2 [41, 42], here represented by strains PAO1 (Table 4) and PA14 (Fig. 5), respectively.

**Table 4.** Determination of MAZ105 pathogenicity in the mouse infection model

Strain	Survival (%)	Abscess area (mm^2^, mean±se) ^†^	Necrotic area^†^ (mm^2^, mean±se) ^†^	Bacterial load	(Log_10_ c.f.u. g^−1^)
				Inoculation area^∗^	Liver^†^
PAO1	41.6	236.5±15.9	29.9±6.7	7.4±0.7 a	2.5±0.7
ATC 9027	100	0**	0**	3.0±0.3 b	0**
PA7	100	0**	0**	2.8±0.3 b	0**
MAZ 105	100	10.5±7**	4.3±2.9**	3.6±0.3 b	0**

Results are the mean of two independent experiments with six mice each. The animals were inoculated subcutaneously with 1012 c.f.u. of the different strains. The abscess area was quantified 24 h after inoculation, and necrotic area at 48 h, which corresponds to the maximum time of its formation. The animals were sacrificed at 96 h; survival and the c.f.u. in the tissues were determined.

*Kruskal–Wallis (p>0.05); Student–Newman–Keuls (α>0.05**).

†ANOVA (*P*<0.05); Tukey (α>0.05).

## Discussion

Strains belonging to *P. aeruginosa* phylogroup 3 have been proposed to constitute a different species (*Pseudomonas paraeruginosa*) due to their genetic distance from strains belonging to other *P. aeruginosa* phylogroups (Fig. 1) [10]. However, even though strains belonging to clade 3 present characteristic genome features, such as deletions of genes involved in T3SS, of *rhlC* that encodes rhamnosyl-transferase II that converts mono- to di-RL and of *phzH* that encodes for the enzyme transforming the PYO precursor phenazine 1-carboxylic acid (PCA) to phenazine 1-carboxamide (PCN), among others [11], the biology of bacteria belonging to this phylogroup is the same as that of members of the other phylogroups. They produce the same infections, possess syntenic chromosomes [43] and use the same regulatory networks to express QS-regulated virulence-associated traits [20, 22]. Furthermore, phylogroups 3 and 5 share the production of ExlA exolysin and the deletion of genes encoding T3SS, RhlC and PhzH, but clade 5 strains contain deletions that originated independently [11] from those of phylogroup 3. Strains belonging to clade 5 are closely related genetically to phylogroups 1 and 2 [7], which encompass the most frequently isolated strains. Thus, strains that belong to the outlier phylogroup 3 are indeed part of the *P. aeruginosa* species, even if genetically distant.

In previous studies [20, 22] we reported that ATCC 9027, a phylogroup 3 strain, contains mutations in *lasR* and *pqsR* but can still produce RL and PYO in phosphate-limited conditions. In this work, we analysed the sequences of 47 genomes of phylogroup 3 strains present in the *Pseudomonas* Genome Database to determine the conservation of QS transcriptional regulators and the frequency of mutations in these genes to obtain a general picture of the QS response in these strains. It is important to note that the RhlR amino acid sequence is highly conserved between all phylogroup 3 strains, emphasizing the central role of this transcriptional regulator in the expression of different virulence-associated traits.

Our bioinformatic analysis showed that loss of function *lasR* mutations are common among *P. aeruginosa* phylogroup 3 strains (Table 3), as has been reported for both clinical and environmental isolates [19]. Naturally occurring *lasR* mutants can express virulence factors and overproduce PYO under phosphate-limited conditions [44], presumably because in these natural mutants *rhlR* is expressed in a LasR-independent manner, as has been reported for some isolates [17–21]. However, these naturally occurring *lasR* mutants generally express low levels of ELA, since full expression of this protease relies on both RhlR and LasR [45]. Thus, the low level of ELA produced by MAZ105 in both LB and FDS media (Fig. 3a) is consistent with it carrying a *lasR* mutation (Table 3).

The analysis of the 47 phylogroup 3 genomes (Table S1) showed that mutations affecting PqsR activity are widespread among these outlier strains (Tables 1 and 2), in contrast to what has been reported for *P. aeruginosa* strains of both clinical and environmental origins that belong to other phylogroups [19]. Furthermore, our bioinformatic analysis (Tables 1–3) and our previously reported results on the characterization of ATCC 9027 LasR protein [20] showed that among the 47 strains studied (Table S1) there are 5 isolates that contain both defective LasR and PqsR proteins. These strains are ATCC 9027 [20, 22], LMG5031, EML528, WH-SG1-V-07064 and MAZ105 (Tables 1–3). Thus, 23 out of the 47 phylogroup 3 strains analysed (Table S1) have mutations affecting the QS response (Tables 1–3), a higher frequency than that reported for other *P. aeruginosa* phylogroups [17–20].

A plausible explanation for the high frequency of mutations affecting the Pqs QS system would be that PqsR in phylogroup 3 strains is inactive and the accumulation of mutations would result from a pseudogene formation process. Our results show that all phylogroup 3 strains contain a phenylalanine at position 201 of PqsR, while strains belonging to other groups contain a serine. This change in position 201 could inactivate PqsR, so the accumulation of further mutations would be a neutral process. However, we show that introducing the F201S change to PAO1 PqsR does not affect the functionality of this protein (Fig. S1).

The in-depth analysis of the MAZ105 phylogroup 3 strain that was isolated from tomato rhizosphere in the Mexican State of Guerrero showed that it contains mutations in *lasR* (Table 3) and *pqsA* (Fig. 2, Table 2), but it can still produce low levels of ELA, RL and PYO under low-phosphate growth conditions (Fig. 3). We showed that MAZ105 is unable to produce PYO in LB medium due to its inability to express *pqsE* from the *pqsA* promoter because of the polar effect of the *pqsA* mutation on the *pqsABCD*E operon expression. However, under low-phosphate conditions, it can produce PYO and transcribe *pqsE* independently of *pqsA* expression (Fig. 4). Thus, we conclude that in this condition *rhlR* is induced, presumably by PhoB as has been shown in the PAO1 strain [21], and that the RhlR interaction with PqsE results in the transcription of genes involved in PYO production.

It has been reported that PqsR ability to activate transcription is strictly dependent on the binding of its co-inducers PQS or HHQ [14], Thus, the *pqsA* mutation in MAZ105 (Fig. 2) and its polar effect on the expression of the *pqsABCDE* operon result in the same phenotype as an inactivating mutation in *pqsR,* which is the lack of PQS and HHQ production (Fig. 3). Therefore, strain MAZ105 contains an inactive PqsR protein even though it lacks mutations in *pqsR*.

The proposed mechanism for PYO production by MAZ105 in low-phosphate medium is similar to the one described for the phylogroup 3 strain ATCC 9027 [22] since in both cases PYO production was independent of the expression of the *pqsABCDE* operon but dependent on *pqsE* expression (Fig. 4). In addition, our bioinformatic analysis of the 47 phylogroup 3 strains showed that another 18 strains besides ATCC 9027 and MAZ105 have a defective Pqs QS system and might have similar *pqsE* expression independently of the *pqsABCDE* operon under some environmental conditions.

The conservation of this molecular mechanism in several independent phylogroup 3 isolates suggests that having a defective Pqs QS system while conserving PYO production in some environmental conditions, such as phosphate limitation, provides them with a selective advantage. Furthermore, these results suggest that in phylogroup 3 strains the QS-regulated virulence traits are strongly modulated by environmental conditions in such a way that the activity of PqsR in expressing the *pqsABCDE* operon is no longer indispensable. The selection of PYO synthesis in strains containing a defective Pqs QS system only under some environmental conditions suggests that high and constitutive PYO production is deleterious for phylogroup 3 strains and might explain the high frequency of *pqsR* mutations in these strains (Table 1), as well as of other defects in the Pqs QS system, such as the *pqsA* mutations in MAZ105, LMG5031 and WH-SGI-V-07287, and the *pqsB* mutation in VFR-PAO1 (Table 2).

Strains belonging to phylogroup 3 are rare among the more than 7000 genomes in the *Pseudomonas* Genome Database (www.pseudomonas.com), since only around 50 genomes of strains belonging to this phylogroup are present [11]. However, this underrepresentation could be due to the prevalence of clinical isolates in this database and their frequency could be higher in other contexts. For example, six out of nine strains of *P. aeruginosa* isolated from healthy farm animals produced ExlA and are thus members of either phylogroups 3 or 5 [46].

Phylogroup 3 strains seem to have attenuated virulence [42], with some exceptions, such as the highly haemolytic CLJ1 strain [38]. We show here that strain MAZ105 is as virulent as PA7 in *G. mellonella* and mouse abscess models, even though it is an environmental isolate. This behaviour highlights that clinical and environmental *P. aeruginosa* isolates constitute a single population [39] and shows that alternative therapeutic strategies not based on antibiotic treatment should target both clinical and environmental strains.

Most anti-virulence treatments proposed to contend with antibiotic-resistant *P. aeruginosa* infections are based on the inactivation of LasR [47] or PqsR [48]. Our results for the high frequency of phylogroup 3 strains with mutations affecting PqsR, LasR or both regulators predict that the use of these anti-QS-based treatments will not be efficient to treat infections of these highly divergent strains. In fact, these treatments might even constitute a further selective pressure for highly antibiotic-resistant strains causing infections, as seems to be the case of PA7 and other highly resistant phylogroup 3 strains that have been isolated from patients, despite their attenuated virulence.

## Supplementary Data

Supplementary material 1
