# Supplementary material for: Pseudomonas aeruginosa strains belonging to phylogroup 3 frequently exhibit an atypical quorum sensing response: the case of MAZ105, a tomato rhizosphere isolate
Source: Microbiology (Reading). 2023 Oct 11;169(10):001401. doi: 10.1099/mic.0.001401 (PMC10634362; doi:10.1099/mic.0.001401)
Supplement: Supplementary material 1 [file mic-169-1401-s001.pdf]

Supplementary information

Title: The quorum sensing response of *Pseudomonas aeruginosa* MAZ105, a tomato-rhizosphere isolate belonging to phylogroup 3.

Authors: Sara E. Quiroz-Morales[1], Luis Felipe Muriel-Millán[2], Gabriel Y. Ponce-Soto[3], Abigail González-Valdez[1], Israel Castillo-Juárez[4], Luis Servín-González[1], Gloria Soberón-Chávez[1]*.

Affiliations: [1]Departamento de Biología Molecular y Biotecnología, Instituto de Investigaciones Biomédicas, Universidad Nacional Autónoma de México, Ciudad Universitaria, Apdo. Postal 70228, C. P. 04510, CDMX, México.

[2]Departamento de Microbiología Molecular, Instituto de Biotecnología, Universidad Nacional Autónoma de México, Av. Universidad 2001, Col. Chamilpa, Cuernavaca, Morelos CP 62210, México.

[3] Microbial Paleogenomics Unit, Department of Genomes & Genetics, Pasteur Institute, 75015 Paris, France.

[4]Laboratorio de Investigación y Aplicación de Fitoquímicos Bioactivos, Colegio de Postgraduados, 56230, Campus Montecillo, Texcoco, México.

*Corresponding author: email address gloria@iibiomedicas.unam.mx

Figure S1. Strain MAZ105 contains genomic regions characteristic of *P. aeruginosa* strains belonging to phylogroup 3 (1). Lanes show the PCR product of a fragment of *exlB*, showing the presence of the *exlBA* operon using either MAZ105 total DNA as template or total DNA from PA7 strain (+); the negative control (-) is the PCR reaction without DNA as template; while the last 2 lanes correspond to the PCR product of the genomic region where *rhlC* is encoded in strains belonging to clades 1 and 2, showing the characteristic deletion of this genes in clade 3 strains.

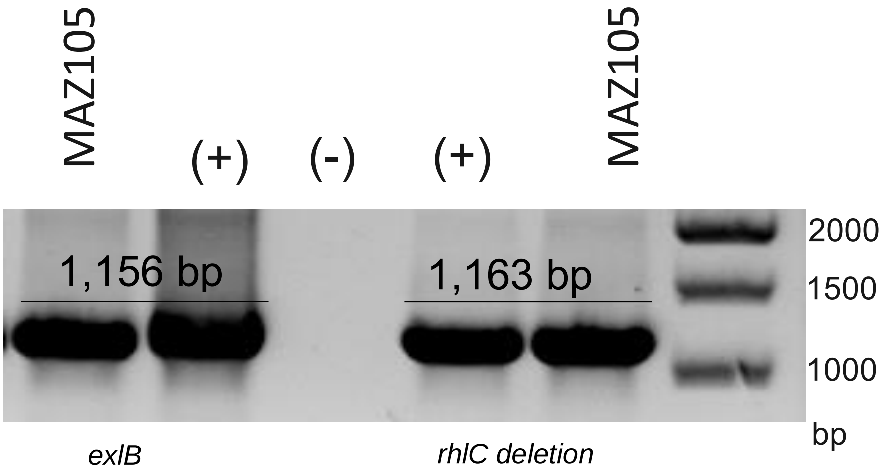

Figure S2. **Multiple sequence alignments of the PAO1 *pqsR* gene and PqsR protein, and of the *lasR* gene and LasR protein.** The sequences of the PAO1 strain were aligned using Clustal Omega with those of several phylogroup 3 strains that contain mutations in the corresponding genes and proteins. Asterisks indicate conserved residues in all sequences. Mutations are highlighted in yellow.

Nucleotide sequence alignment of *pqs*R

```
CLUSTAL O(1.2.4) multiple sequence alignment


PAO1                ATGCCTATTCATAACCTGAATCACGTGAACATGTTCCTCCAGGTCATCGCCTCCGGTTCG     60
ATCC9027            ATGCCTATTCATAACCTCAATCACGTCAACATGTTCCTCCAGGTCATCGCCTCCGGCTCG     60
ATCC33359           ATGCCTATTCATAACCTCAATCACGTCAACATGTTCCTCCAGGTCATCGCCTCCGGCTCG     60
EML545              ATGCCTATTCATAACCTCAATCACGTCAACATGTTCCTCCAGGTCATCGCCTCCGGCTCG     60
AR_0356             ATGCCTATTCATAACCTCAATCACGTCAACATGTTCCTCCAGGTCATCGCCTCCGGCTCG     60
AR441               ATGCCTATTCATAACCTCAATCACGTCAACATGTTCCTCCAGGTCATCGCCTCCGGCTCG     60
WH-SGI-V-07165      ATGCCTATTCATAACCTCAATCACGTCAACATGTTCCTCCAGGTCATCGCCTCCGGCTCG     60
EML1796             ATGCCTATTCATAACCTCAATCACGTCAACATGTTCCTCCAGGTCATCGCCTCCGGCTCG     60
EML1795             ATGCCTATTCATAACCTCAATCACGTCAACATGTTCCTCCAGGTCATCGCCTCCGGCTCG     60
MIN-137             ATGCCTATTCATAACCTCAATCACGTCAACATGTTCCTCCAGGTCATCGCCTCCGGCTCG     60
PSA00358            ATGCCTATTCATAACCTCAATCACGTCAACATGTTCCTCCAGGTCATCGCCTCCGGCTCG     60
PSA00289            ATGCCTATTCATAACCTCAATCACGTCAACATGTTCCTCCAGGTCATCGCCTCCGGCTCG     60
PA7                 ATGCCTATTCATAACCTCAATCACGTCAATATGTTCCTCCAGGTCATCGCCTCCGGCTCG     60
PABL043             ATGCCTATTCATAACCTCAATCACGTCAATATGTTCCTCCAGGTCATCGCCTCCGGCTCG     60
WH-SGI-V-07064      ATGCCTATTCATAACCTCAATCACGTCAATATGTTCCTCCAGGTCATCGCCTCCGGCTCG     60
WH-SGI-V-07072      ATGCCTATTCATAACCTCAATCACGTCAATATGTTCCTCCAGGTCATCGCCTCCGGCTCG     60
MAZ105              ATGCCTATTCATAACCTCAATCACGTCAATATGTTCCTCCAGGTCATCGCCTCCGGCTCG     60
WH-SGI-V-07370      ATGCCTATTCATAACCTCAATCACGTCAATATGTTCCTCCAGGTCATCGCCTCCGGCTCG     60
                    ***************** ******** ** ************************** ***

PAO1                ATTTCCTCCGCTGCGCGGATCCTGCGCAAGTCGCACACCGCGGTCAGCTCGGCGGTCAGC     120
ATCC9027            ATCTCTTCCGCCGCGCGTATCCTGCGCAAGTCGCACACCGCGGTCAGCTCGGCGGTGAGC     120
ATCC33359           ATCTCTTCCGCCGCGCGTATCCTGCGCAAGTCGCACACCGCGGTCAGCTCGGCGGTGAGC     120
EML545              ATCTCTTCCGCCGCGCGTATCCTGCGCAAGTCGCACACCGCGGTCAGCTCGGCGGTGAGC     120
AR_0356             ATCTCTTCCGCTGCGCGTATCCTGCGCAAGTCGCACACCGCGGTCAGCTCGGCGGTGAGC     120
AR441               ATCTCTTCCGCTGCGCGTATCCTGCGCAAGTCGCACACCGCGGTCAGCTCGGCGGTGAGC     120
WH-SGI-V-07165      ATCTCTTCCGCTGCGCGTATCCTGCGCAAGTCGCACACCGCGGTCAGCTCGGCGGTGAGC     120
EML1796             ATCTCTTCCGCTGCGCGTATCCTGCGCAAGTCGCACACCGCGGTCAGCTCGGCGGTGAGC     120
EML1795             ATCTCTTCCGCTGCGCGTATCCTGCGCAAGTCGCACACCGCGGTCAGCTCGGCGGTGAGC     120
MIN-137             ATCTCTTCCGCTGCGCGTATCCTGCGCAAGTCGCACACCGCGGTCAGCTCGGCGGTGAGC     120
PSA00358            ATCTCTTCCGCTGCGCGTATCCTGCGCAAGTCGCACACCGCGGTCAGCTCGGCGGTGAGC     120
PSA00289            ATCTCTTCCGCTGCGCGTATCCTGCGCAAGTCGCACACCGCGGTCAGCTCGGCGGTGAGC     120
PA7                 ATCTCTTCCGCCGCGCGTATCCTGCGCAAGTCGCACACCGCGGTCAGCTCGGCGGTCAGC     120
PABL043             ATCTCTTCCGCCGCGCGTATCCTGCGCAAGTCGCACACCGCGGTCAGCTCGGCGGTCAGC     120
WH-SGI-V-07064      ATCTCTTCCGCCGCGCGTATCCTGCGCAAGTCGCACACCGCGGTCAGCTCGGCGGTCAGC     120
WH-SGI-V-07072      ATCTCTTCCGCCGCGCGTATCCTGCGCAAGTCGCACACCGCGGTCAGCTCGGCGGTCAGC     120
MAZ105              ATCTCTTCCGCCGCGCGTATCCTGCGCAAGTCGCACACCGCGGTCAGCTCGGCGGTCAGC     120
WH-SGI-V-07370      ATCTCTTCCGCCGCGCGTATCCTGCGCAAGTCGCACACCGCGGTCAGCTCGGCGGTCAGC     120
                    ** ** ***** ***** ************************************** ***

PAO1                AACCTGGAAATCGACCTGTGCGTGGAGCTGGTCCGTCGGGACGGCTACAAGGTCGAACCC     180
ATCC9027            AACCTGAAAATCGACCTGTGCGTGGAGCTGGTCCGTCGCGACGGCTACAAGGTCGAACCC     180
ATCC33359           AACCTGGAAATCGACCTGTGCGTGGAGCTGGTCCGTCGCGACGGCTACAAGGTCGAACCC     180
EML545              AACCTGGAAATCGACCTGTGCGTGGAGCTGGTCCGTCGCGACGGCTACAAGGTCGAACCC     180
AR_0356             AACCTGGAAATCGACCTGTGCGTGGAGCTGGTCCGTCGCGACGGCTACAAGGTCGAACCC     180
AR441               AACCTGGAAATCGACCTGTGCGTGGAGCTGGTCCGTCGCGACGGCTACAAGGTCGAACCC     180
WH-SGI-V-07165      AACCTGGAAATCGACCTGTGCGTGGAGCTGGTCCGTCGCGACGGCTACAAGGTCGAACCC     180
EML1796             AACCTGGAAATCGACCTGTGCGTGGAGCTGGTCCGTCGCGACGGCTACAAGGTCGAACCC     180
EML1795             AACCTGGAAATCGACCTGTGCGTGGAGCTGGTCCGTCGCGACGGCTACAAGGTCGAACCC     180
MIN-137             AACCTGGAAATCGACCTGTGCGTGGAGCTGGTCCGTCGCGACGGCTACAAGGTCGAACCC     180
PSA00358            AACCTGGAAATCGACCTGTGCGTGGAGCTGGTCCGTCGCGACGGCTACAAGGTCGAACCC     180
PSA00289            AACCTGGAAATCGACCTGTGCGTGGAGCTGGTCCGTCGCGACGGCTACAAGGTCGAACCC     180
PA7                 AACCTGGAAATCGACCTGTGCGTGGAGCTGGTCCGTCGCGACGGCTACAAGGTCGAACCC     180
PABL043             AACCTGGAAATCGACCTGTGCGTGGAGCTGGTCCGTCGCGACGGCTACAAGGTCGAACCC     180
```

```
WH-SGI-V-07064     AACCTGGAAATCGACCTGTGCGTGGAGCTGGTCCGTCGCGACGGCTACAAGGTCGAACCC     180
WH-SGI-V-07072     AACCTGGAAATCGACCTGTGCGTGGAGCTGGTCCGTCGCGACGGCTACAAGGTCGAACCC     180
MAZ105             AACCTGGAAATCGACCTGTGCGTGGAGCTGGTCCGTCGCGACGGCTACAAGGTCGAACCC     180
WH-SGI-V-07370     AACCTGGAAATCGACCTGTGCGTGGAGCTGGTCCGTCGCGACGGCTACAAGGTCGAACCC     180
                   ****** ******************************* *********************

PAO1               ACCGAGCAGGCGCTTCGCCTGATCCCTTACATGCGCAGCCTGCTGAACTACCAGCAGCTG     240
ATCC9027           ACCGAGCAGGCGCTACGCCTGATCCCCTACATGCGCAGCCTGCTGAACTACCAGCAGCTG     240
ATCC33359          ACCGAGCAGGCGCTACGCCTGATCCCCTACATGCGCAGCCTGCTGAACTACCAGCAGCTG     240
EML545             ACCGAGCAGGCGCTACGCCTGATCCCCTACATGCGCAGCCTGCTGAACTACCAGCAGCTG     240
AR_0356            ACCGAGCAGGCGCTACGCCTGATCCCCTACATGCGCAGCCTGCTGAACTACCAGCAGCTG     240
AR441              ACCGAGCAGGCGCTACGCCTGATCCCCTACATGCGCAGCCTGCTGAACTACCAGCAGCTG     240
WH-SGI-V-07165     ACCGAGCAGGCGCTACGCCTGATCCCCTACATGCGCAGCCTGCTGAACTACCAGCAGCTG     240
EML1796            ACCGAGCAGGCGCTACGCCTGATCCCCTACATGCGCAGCCTGCTGAACTACCAGCAGCTG     240
EML1795            ACCGAGCAGGCGCTACGCCTGATCCCCTACATGCGCAGCCTGCTGAACTACCAGCAGCTG     240
MIN-137            ACCGAGCAGGCGCTACGCCTGATCCCCTACATGCGCAGCCTGCTGAACTACCAGCAGCTG     240
PSA00358           ACCGAGCAGGCGCTACGCCTGATCCCCTACATGCGCAGCCTGCTGAACTACCAGCAGCTG     240
PSA00289           ACCGAGCAGGCGCTACGCCTGATCCCCTACATGCGCAGCCTGCTGAACTACCAGCAGCTG     240
PA7                ACCGAGCAGGCGCTACGCCTGATCCCTTACATGCGCAGCCTGCTGAACTACCAGCAGCTG     240
PABL043            ACCGAGCAGGCGCTACGCCTGATCCCTTACATGCGCAGCCTGCTGAACTACCAGCAGCTG     240
WH-SGI-V-07064     ACCGAGCAGGCGCTACGCCTGATCCCTTACATGCGCAGCCTGCTGAACTACCAGCAGCTG     240
WH-SGI-V-07072     ACCGAGCAGGCGCTACGCCTGATCCCTTACATGCGCAGCCTGCTGAACTACCAGCAGCTG     240
MAZ105             ACCGAGCAGGCGCTACGCCTGATCCCTTACATGCGCAGCCTGCTGAACTACCAGCAGCTG     240
WH-SGI-V-07370     ACCGAGCAGGCGCTACGCCTGATCCCTTACATGCGCAGCCTGCTGAACTACCAGCAGCTG     240
                   ************** *********** *********************************

PAO1               ATCGGCGACATCGCCTTCAATCTCAACAAGGGTCCGCGCAATCTCCGGGTGCTGCTGGAC     300
ATCC9027           ATCGGCGACATCGCCTTCAACCTCAACAAGGGGCCACGCAACCTGCGGGTGCTGCTGGAT     300
ATCC33359          ATCGGCGACATCGCCTTCAACCTCAACAAGGGGCCACGCAACCTGCGGGTGCTGCTGGAT     300
EML545             ATCGGCGACATCGCCTTCAACCTCAACAAGGGGCCACGCAACCTGCGGGTGCTGCTGGAT     300
AR_0356            ATCGGCGACATCGCCTTCAACCTCAACAAGGGGCCACGCAACCTGCGGGTGCTGCTGGAT     300
AR441              ATCGGCGACATCGCCTTCAACCTCAACAAGGGGCCACGCAACCTGCGGGTGCTGCTGGAT     300
WH-SGI-V-07165     ATCGGCGACATCGCCTTCAACCTCAACAAGGGGCCACGCAACCTGCGGGTGCTGCTGGAT     300
EML1796            ATCGGCGACATCGCCTTCAACCTCAACAAGGGGCCACGCAACCTGCGGGTGCTGCTGGAT     300
EML1795            ATCGGCGACATCGCCTTCAACCTCAACAAGGGGCCACGCAACCTGCGGGTGCTGCTGGAT     300
MIN-137            ATCGGCGACATCGCCTTCAACCTCAACAAGGGGCCACGCAACCTGCGGGTGCTGCTGGAT     300
PSA00358           ATCGGCGACATCGCCTTCAACCTCAACAAGGGGCCACGCAACCTGCGGGTGCTGCTGGAT     300
PSA00289           ATCGGCGACATCGCCTTCAACCTCAACAAGGGGCCACGCAACCTGCGGGTGCTGCTGGAT     300
PA7                ATCGGCGACATCGCCTTCAACCTCAACAAGGGGCCGCGCAACCTGCGGGTGCTGCTGGAT     300
PABL043            ATCGGCGACATCGCCTTCAACCTCAACAAGGGGCCGCGCAACCTGCGGGTGCTGCTGGAT     300
WH-SGI-V-07064     ATCGGCGACATCGCCTTCAACCTCAACAAGGGGCCGCGCAACCTGCGGGTGCTGCTGGAT     300
WH-SGI-V-07072     ATCGGCGACATCGCCTTCAACCTCAACAAGGGGCCGCGCAACCTGCGGGTGCTGCTGGAT     300
MAZ105             ATCGGCGACATCGCCTTCAACCTCAACAAGGGGCCGCGCAACCTGCGGGTGCTGCTGGAT     300
WH-SGI-V-07370     ATCGGCGACATCGCCTTCAACCTCAACAAGGGGCCGCGCAACCTGCGGGTGCTGCTGGAT     300
                   ******************** *********** ** ***** ** **************

PAO1               ACCGCCATCCCGCCGTCGTTCTGCGATACGGTGAGCAGCGTACTGCTCGACGATTTCAAC     360
ATCC9027           ACGGCAATCCCGCCATCGTTCTGCGACACGGTGAGCAGCGTCCTGCTCGACGACTTCAAC     360
ATCC33359          ACGGCAATCCCGCCATCGTTCTGCGACACGGTGAGCAGCGTCCTGCTCGACGACTTCAAC     360
EML545             ACGGCAATCCCGCCATCGTTCTGCGACACGGTGAGCAGCGTCCTGCTCGACGACTTCAAC     360
AR_0356            ACGGCAATCCCGCCATCGTTCTGCGACACGGTGAGCAGCGTCCTGCTCGACGACTTCAAC     360
AR441              ACGGCAATCCCGCCATCGTTCTGCGACACGGTGAGCAGCGTCCTGCTCGACGACTTCAAC     360
WH-SGI-V-07165     ACGGCAATCCCGCCATCGTTCTGCGACACGGTGAGCAGCGTCCTGCTCGACGACTTCAAC     360
EML1796            ACGGCAATCCCGCCATCGTTCTGCGACACGGTGAGCAGCGTCCTGCTCGACGACTTCAAC     360
EML1795            ACGGCAATCCCGCCATCGTTCTGCGACACGGTGAGCAGCGTCCTGCTCGACGACTTCAAC     360
MIN-137            ACGGCAATCCCGCCATCGTTCTGCGACACGGTGAGCAGCGTCCTGCTCGACGACTTCAAC     360
PSA00358           ACGGCAATCCCGCCATCGTTCTGCGACACGGTGAGCAGCGTCCTGCTCGACGACTTCAAC     360
PSA00289           ACGGCAATCCCGCCATCGTTCTGCGACACGGTGAGCAGCGTCCTGCTCGACGACTTCAAC     360
PA7                ACGGCAATCCCGCCATCGTTCTGCGACACGGTGAGCAGCGTCCTGCTCGACGACTTCAAC     360
PABL043            ACGGCAATCCCGCCATCGTTCTGCGACACGGTGAGCAGCGTCCTGCTCGACGACTTCAAC     360
WH-SGI-V-07064     ACGGCAATCCCGCCATCGTTCTGCGACACGGTGAGCAGCGTCCTGCTCGACGACTTCAAC     360
WH-SGI-V-07072     ACGGCAATCCCGCCATCGTTCTGCGACACGGTGAGCAGCGTCCTGCTCGACGACTTCAAC     360
MAZ105             ACGGCAATCCCGCCATCGTTCTGCGACACGGTGAGCAGCGTCCTGCTCGACGACTTCAAC     360
WH-SGI-V-07370     ACGGCAATCCCGCCATCGTTCTGCGACACGGTGAGCAGCGTCCTGCTCGACGACTTCAAC     360
                   ** ** ******** *********** ************** *********** ******
```

```
PAO1                ATGGTCAGCCTGATACGCACCTCGCCCGCCGATAGCCTGGCGACGATCAAGCAGGACAAC    420
ATCC9027            ATGGTCAGCCTGATACGCACCTCGCCGGCCGACAGCCTGGCGACCATCAAGCAGGACAAC    420
ATCC33359           ATGGTCAGCCTGATACGCACCTCGCCGGCCGACAGCCTGGCGACCATCAAGCAGGACAAC    420
EML545              ATGGTCAGCCTGATACGCACCTCGCCGGCCGACAGCCTGGCGACCATCAAGCAGGACAAC    420
AR_0356             ATGGTCAGCCTGATACGCACCTCGCCGGCCGACAGCCTGGCGACCATCAAGCAGGACAAC    420
AR441               ATGGTCAGCCTGATACGCACCTCGCCGGCCGACAGCCTGGCGACCATCAAGCAGGACAAC    420
WH-SGI-V-07165      ATGGTCAGCCTGATACGCACCTCGCCGGCCGACAGCCTGGCGACCATCAAGCAGGACAAC    420
EML1796             ATGGTCAGCCTGATACGCACCTCGCCGGCCGACAGCCTGGCGACCATCAAGCAGGACAAC    420
EML1795             ATGGTCAGCCTGATACGCACCTCGCCGGCCGACAGCCTGGCGACCATCAAGCAGGACAAC    420
MIN-137             ATGGTCAGCCTGATACGCACCTCGCCGGCCGACAGCCTGGCGACCATCAAGCAGGACAAC    420
PSA00358            ATGGTCAGCCTGATACGCACCTCGCCGGCCGACAGCCTGGCGACCATCAAGTAGGACAAC    420
PSA00289            ATGGTCAGCCTGATACGCACCTCGCCGGCCGACAGCCTGGCGACCATCAAGTAGGACAAC    420
PA7                 ATGGTCAGCCTGATACGCACCTCGCCGGCCGACAGCCTGGCGACCATCAAGCAGGACAAC    420
PABL043             ATGGTCAGCCTGATACGCACCTCGCCGGCCGACAGCCTGGCGACCATCAAGCAGGACAAC    420
WH-SGI-V-07064      ATGGTCAGCCTGATACGCACCTCGCCGGCCGACAGCCTGGCGACCATCAAGCAGGACAAC    420
WH-SGI-V-07072      ATGGTCAGCCTGATACGCACCTCGCCGGCCGACAGCCTGGCGACCATCAAGCAGGACAAC    420
MAZ105              ATGGTCAGCCTGATACGCACCTCGCCGGCCGACAGCCTGGCGACCATCAAGCAGGACAAC    420
WH-SGI-V-07370      ATGGTCAGCCTGATACGCACCTCGCCGGCCGACAGCCTGGCGACCATCAAGCAGGACAAC    420
                    ************************** ***** *********** ****** ********

PAO1                GCGGAAATCGATATCGCCATCACCATCGACGAGGAACTGAAGATCTCCCGCTTCAACCAG    480
ATCC9027            GCGGAAATCGACATCGCCATCACCATCGACGAGGAGTTGAAGATTTCCCGCTTCAACCAG    480
ATCC33359           GCGGAAATCGACATCG------CCATCGACGAGGAGTTGAAGATCTCCCGCTTCAACCAG    474
EML545              GCGGAAATCGACATC------GCCATCGACGAGGAGTTGAAGATCTCCCGCTTCAACCAG    474
AR_0356             GCGGAAATCGACATCGCCATCACCATC---------------ATCTCCCGCTTCAACCAG    465
AR441               GCGGAAATCGACATCGCCATCACCATC---------------ATCTCCCGCTTCAACCAG    465
WH-SGI-V-07165      GCGGAAATCGACATCGCCATCACCATC---------------ATCTCCCGCTTCAACCAG    465
EML1796             GCGGAAATCGACATCGCCATCACCATC---------------ATCTCCCGCTTCAACCAG    465
EML1795             GCGGAAATCGACATCGCCATCACCATC---------------ATCTCCCGCTTCAACCAG    465
MIN-137             GCGGAAATCGACATCGCCATCACCATCGACGAGGAGTTGAAGATCTCCCGCTTCAACCAG    480
PSA00358            GCGGAAATCGACATCGCCATCACCATCGACGAGGAGTTGAAGATCTCCCGCTTCAACCAG    480
PSA00289            GCGGAAATCGACATCGCCATCACCATCGACGAGGAGTTGAAGATCTCCCGCTTCAACCAG    480
PA7                 GCGGAAATCGACATCGCCATCACCATCGACGAGGAGTTGAAGATCTCCCGCTTCAACCAG    480
PABL043             GCGGAAATCGACATCGCCATCACCATCGACGAGGAGTTGAAGATCTCCCGCTTCAACCAG    480
WH-SGI-V-07064      GCGGAAATCGACATCGCCATCACCATCGACGAGGAGTTGAAGATCTCCCGCTTCAACCAG    480
WH-SGI-V-07072      GCGGAAATCGACATCGCCATCACCATCGACGAGGAGTTGAAGATCTCCCGCTTCAACCAG    480
MAZ105              GCGGAAATCGACATCGCCATCACCATCGACGAGGAGTTGAAGATCTCCCGCTTCAACCAG    480
WH-SGI-V-07370      GCGGAAATCGACATCGCCATCACCATCGACGAGGAGTTGAAGATCTCCCGCTTCAACCAG    480
                    *********** ***       *****               ** ***************

PAO1                TGCGTGCTCGGCTACACCAAGGCGTTCGTCGTCGCCCATCCGCAGCACCCGTTGTGCAAT    540
ATCC9027            TGCGTGCTCGGCTACACCAAGGCGTTCGTCGTCGCCCATCCCCAGCATCCGCTGTGCAAT    540
ATCC33359           TGCGTGCTCGGCTACACCAAGGCGTTCGTCGTCGCCCATCCCCAGCATCCGCTGTGCAAT    534
EML545              TGCGTGCTCGGCTACACCAAGGCGTTCGTCGTCGCCCATCCCCAGCATCCGCTGTGCAAT    534
AR_0356             TGCGTGCTCGGCTACACCAAGGCGTTCGTCGTCGCCCACCCCCAGCATCCGCTGTGCAAT    525
AR441               TGCGTGCTCGGCTACACCAAGGCGTTCGTCGTCGCCCACCCCCAGCATCCGCTGTGCAAT    525
WH-SGI-V-07165      TGCGTGCTCGGCTACACCAAGGCGTTCGTCGTCGCCCACCCCCAGCATCCGCTGTGCAAT    525
EML1796             TGCGTGCTCGGCTACACCAAGGCGTTCGTCGTCGCCCACCCCCAGCATCCGCTGTGCAAT    525
EML1795             TGCGTGCTCGGCTACACCAAGGCGTTCGTCGTCGCCCACCCCCAGCATCCGCTGTGCAAT    525
MIN-137             TGCGTGCTCGGCTACACCAAGGCGTTCGTCGTCGCCCATCCCCAGCATCCGCTGTGCAAT    540
PSA00358            TGCGTGCTCGGCTACACCAAGGCGTTCGTCGTCGCCCATCCCCAGCATCCGCTGTGCAAT    540
PSA00289            TGCGTGCTCGGCTACACCAAGGCGTTCGTCGTCGCCCATCCCCAGCATCCGCTGTGCAAT    540
PA7                 TGCGTGCTCGGCTACACCAAGGCGTTCGTCGTCGCCCATCCCCAGCATCCGCTGTGCAAT    540
PABL043             TGCGTGCTCGGCTACACCAAGGCGTTCGTCGTCGCCCATCCCCAGCATCCGCTGTGCAAT    540
WH-SGI-V-07064      TGCGTGCTCGGCTACACCAAGGCGTTCGTCGTCGCCCATCCCCAGCATCCGCTGTGCAAT    540
WH-SGI-V-07072      TGCGTGCTCGGCTACACCAAGGCGTTCGTCGTCGCCCATCCCCAGCATCCGCTGTGCAAT    540
MAZ105              TGCGTGCTCGGCTACACCAAGGCGTTCGTCGTCGCCCATCCCCAGCATCCGCTGTGCAAT    540
```

```
WH-SGI-V-07370      TGCGTGCTCGGCTACACCAAGGCGTTCGTCGCCCATCCCCAGCATCCGCTGTGCAAT     540
                    ************************************** ** ***** *** ********

PAO1                GCCTCCCTGCAC---AGCATCGCGAGCCTGGCCAATTACCGGCAGATCAGCCTCGGCAGC     597
ATCC9027            GCTTCGCTGCAC---AGCATCGCGAGCCTGGCCAACTACCGGCAGATCAGCCTCGGCAGC     597
ATCC33359           GCTTCGCTGCAC---AGCATCGCGAGCCTGGCCAACTACCGGCAGATCAGCCTCGGCAGC     591
EML545              GCTTCGCTGCAC---AGCATCGCGAGCCTGGCCAACTACCGGCAGATCAGCCTCGGCAGC     591
AR_0356             GCTTCGCTGCAC---AGCATCGCGAGCCTGGCCAACTACCGGCAGATCAGCCTCGGCAGC     582
AR441               GCTTCGCTGCAC---AGCATCGCGAGCCTGGCCAACTACCGGCAGATCAGCCTCGGCAGC     582
WH-SGI-V-07165      GCTTCGCTGCAC---AGCATCGCGAGCCTGGCCAACTACCGGCAGATCAGCCTCGGCAGC     582
EML1796             GCTTCGCTGCAC---AGCATCGCGAGCCTGGCCAACTACCGGCAGATCAGCCTCGGCAGC     582
EML1795             GCTTCGCTGCAC---AGCATCGCGAGCCTGGCCAACTACCGGCAGATCAGCCTCGGCAGC     582
MIN-137             GCTTCGCTGCAC---AGCATCGCGAGCCTGGCCAACTACCGGCAGATCAGCCTCGGCAGC     597
PSA00358            GCTTCGCTGCAC---AGCATCGCGAGCCTGGCCAACTACCGGCAGATCAGCCTCGGCAGC     597
PSA00289            GCTTCGCTGCAC---AGCATCGCGAGCCTGGCCAACTACCGGCAGATCAGCCTCGGCAGC     597
PA7                 GCCTCGCTGCAC---AGCATAGCGAGCCTGGCCAACTACCGGCAGATCAGCCTCGGCAGC     597
PABL043             GCCTCGCTGCAC---AGCATAGCGAGCCTGGCCAACTACCGGCAGATCAGCCTCGGCAGC     597
WH-SGI-V-07064      GCCTCGCTGCAC---AGCATAGCGAGCCTGGCCAACTACCGGCAGATCAGCCTCGGCAGC     597
WH-SGI-V-07072      GCCTCGCTGCAC---AGCATAGCGAGCCTGGCCAACTACCGGCAGATCAGCCTCGGCAGC     597
MAZ105              GCCTCGCTGCAC---AGCATAGCGAGCCTGGCCAACTACCGGCAGATCAGCCTCGGCAGC     597
WH-SGI-V-07370      GCCTCGCTGCACAACAGCATAGCGAGCCTGGCCAACTACCGGCAGATCAGCCTCGGCAGC     600
                    ** ** ******   ***** ************** ************************

PAO1                CGCTCCGGGCAGCATTCGAACCTGCTGCGGCCGGTCAGCGACAAGGTGCTCTTCGTGGAA     657
ATCC9027            CGCTTCGGCCAGCATTCGAACCTGCTTCGCCCGGTCAGCGACAAGGTGCTCTTCGTGGAG     657
ATCC33359           CGCTTCGGCCAGCATTCGAACCTGCTTCGCCCGGTCAGCGACAAGGTGCTCTTCGTGGAG     651
EML545              CGCTTCGGCCAGCATTCGAACCTGCTTCGCCCGGTCAGCGACAAGGTGCTCTTCGTGGAG     651
AR_0356             CGCTTCGGCCAGCATTCGAACCTGCTTCGCCCGGTCAGCGACAAGGTGCTCTTCGTGGAG     642
AR441               CGCTTCGGCCAGCATTCGAACCTGCTTCGCCCGGTCAGCGACAAGGTGCTCTTCGTGGAG     642
WH-SGI-V-07165      CGCTTCGGCCAGCATTCGAACCTGCTTCGCCCGGTCAGCGACAAGGTGCTCTTCGTGGAG     642
EML1796             CGCTTCGGCCAGCATTCGAACCTGCTTCGCCCGGTCAGCGACAAGGTGCTCTTCGTGGAG     642
EML1795             CGCTTCGGCCAGCATTCGAACCTGCTTCGCCCGGTCAGCGACAAGGTGCTCTTCGTGGAG     642
MIN-137             CGCTTCGGCCAGCATTCGAACCTGCTTCGCCCGGTCAGCGACAAGGTGCTCTTCGTGGAG     657
PSA00358            CGCTTCGGCCAGCATTCGAACCTGCTTCGCCCGGTCAGCGACAAGGTGCTCTTCGTGGAG     657
PSA00289            CGCTTCGGCCAGCATTCGAACCTGCTTCGCCCGGTCAGCGACAAGGTGCTCTTCGTGGAG     657
PA7                 CGCTTCGGCCAGCATTCGAACCTGCTTCGCCCGGTCAGCGACAAGGTGCTCTTCGTGGAG     657
PABL043             CGCTTCGGCCAGCATTCGAACCTGCTTCGCCCGGTCAGCGACAAGGTGCTCTTCGTGGAG     657
WH-SGI-V-07064      CGCTTCGGCCAGCATTCGAACCTGCTTCGCCCGGTCAGCGACAAGGTGCTCTTCGTGGAG     657
WH-SGI-V-07072      CGCTTCGGCCAGCATTCGAACCTGCTTCGCCCGGTCAGCGACAAGGTGCTCTTCGTGGAG     657
MAZ105              CGCTTCGGCCAGCATTCGAACCTGCTTCGCCCGGTCAGCGACAAGGTGCTCTTCGTGGAG     657
WH-SGI-V-07370      CGCTTCGGCCAGCATTCGAACCTGCTTCGCCCGGTCAGCGACAAGGTGCTCTTCGTGGAG     660
                    **** *** ***************** ** *****************************

PAO1                AACTTCGACGACATGCTGCGTCTGGTGGAAGCCGGCGTCGGATGGGGCATCGCGCCGCAT     717
ATCC9027            AACTTCGACGATATGCTGCGCCTGGTGGAAGCCGGCGTGGGCTGGGGCATCGCGCCGCAC     717
ATCC33359           AACTTCGACGATATGCTGCGCCTGGTGGAAGCCGGCGTGGGCTGGGGCATCGCGCCGCAC     711
EML545              AACTTCGACGATATGCTGCGCCTGGTGGAAGCCGGCGTGGGCTGGGGCATCGCGCCGCAC     711
AR_0356             AACTTCGACGATATGCTGCGCCTGGTGGAAGCCGGCGTGGGCTGGGGCATCGCGCCGCAC     702
AR441               AACTTCGACGATATGCTGCGCCTGGTGGAAGCCGGCGTGGGCTGGGGCATCGCGCCGCAC     702
WH-SGI-V-07165      AACTTCGACGATATGCTGCGCCTGGTGGAAGCCGGCGTGGGCTGGGGCATCGCGCCGCAC     702
EML1796             AACTTCGACGATATGCTGCGCCTGGTGGAAGCCGGCGTGGGCTGGGGCATCGCGCCGCAC     702
EML1795             AACTTCGACGATATGCTGCGCCTGGTGGAAGCCGGCGTGGGCTGGGGCATCGCGCCGCAC     702
MIN-137             AACTTCGACGATATGCTGCGCCTGGTGGAAGCCGGCGTGGG-------------CCGCAC     704
PSA00358            AACTTCGACGATATGCTGCGCCTGGTGGAAGCCGGCGTGGGCTGGGGCATCGCGCCGCAC     717
PSA00289            AACTTCGACGATATGCTGCGCCTGGTGGAAGCCGGCGTGGGCTGGGGCATCGCGCCGCAC     717
PA7                 AACTTCGACGATATGCTGCGCCTGGTGGAAGCCGGCGTGG--TGGGGTATCGCGCCGCAC     715
PABL043             AACTTCGACGATATGCTGCGCCTGGTGGAAGCCGGCGTGG--TGGGGTATCGCGCCGCAC     715
WH-SGI-V-07064      AACTTCGACGATATGCTGCGCCTGGTGGAAGCCGGCGTGG--TGGGGTATCGCGCCGCAC     715
WH-SGI-V-07072      AACTTCGACGATATGCTGCGCCTGGTGGAAGCCGGCGTGG--TGGGGTATCGCGCCGCAC     715
MAZ105              AACTTCGACGATATGCTGCGCCTGGTGGAAGCCGGCGTGG--TGGGGTATCGCGCCGCAC     715
WH-SGI-V-07370      AACTTCGACGATATGCTGCGCCTGGTGGAAGCCGGCGTGGGCTGGGGTATCGCGCCGCAC     720
                    *********** ******** ***************** *              *****

PAO1                TATTTCGTCGAGGAACGCCTGCGCAACGGTACCCT-GGCAGTCCTCAGCGAACTCTACGA     776
ATCC9027            TATTTCGTCGAGGAGCGCCTGCGCGCCGGTACCCTGGGCCGTTCTCAGCGAGCTGTACGA     777
ATCC33359           TATTTCGTCGAGGAGCGCCTGCGCGCCGGTACCCTG-GCCGTTCTCAGCGAGCTGTACGA     770
EML545              TATTTCGTCGAGGAGCGCCTGCGCGCCGGTACCCTG-GCCGTTCTCAGCGAGCTGTACGA     770
AR_0356             TATTTCGTCGAGGAGCGCCTGCGCGCCGGTACCCTG-GCCGTTCTCAGCGAGCTGTACGA     761
AR441               TATTTCGTCGAGGAGCGCCTGCGCGCCGGTACCCTG-GCCGTTCTCAGCGAGCTGTACGA     761
```

```
WH-SGI-V-07165     TATTTCGTCGAGGAGCGCCTGCGCGCCGGTACCCTG-GCCGTTCTCAGCGAGCTGTACGA     761
EML1796            TATTTCGTCGAGGAGCGCCTGCGCGCCGGTACCCTG-GCCGTTCTCAGCGAGCTGTACGA     761
EML1795            TATTTCGTCGAGGAGCGCCTGCGCGCCGGTACCCTG-GCCGTTCTCAGCGAGCTGTACGA     761
MIN-137            TATTTCGTCGAGGAGCGCCTGCGCGCCGGTACCCTG-GCCGTTCTCAGCGAGCTGTACGA     763
PSA00358           TATTTCGTCGAGGAGCGCCTGCGCGCCGGTACCCTG-GCCGTTCTCAGCGAGCTGTACGA     776
PSA00289           TATTTCGTCGAGGAGCGCCTGCGCGCCGGTACCCTG-GCCGTTCTCAGCGAGCTGTACGA     776
PA7                TATTTCGTCGAGGAGCGCCTACGCACCGGCACCCT-GGCCGTTCTCAGCGAGCTGTACGA     774
PABL043            TATTTCGTCGAGGAGCGCCTACGCACCGGCACCCT-GGCCGTTCTCAGCGAGCTGTACGA     774
WH-SGI-V-07064     TATTTCGTCGAGGAGCGCCTACGCACCGGCACCCT-GGCCGTTCTCAGCGAGCTGTACGA     774
WH-SGI-V-07072     TATTTCGTCGAGGAGCGCCTACGCACCGGCACCCT-GGCCGTTCTCAGCGAGCTGTACGA     774
MAZ105             TATTTCGTCGAGGAGCGCCTACGCACCGGCACCCT-GGCCGTTCTCAGCGAGCTGTACGA     774
WH-SGI-V-07370     TATTTCGTCGAGGAGCGCCTACGCACCGGCACCCT-GGCCGTTCTCAGCGAGCTGTACGA     779
                   ************** ***** ***  *** *****  ** ** ******** ** *****

PAO1               ACCGGGCGGCATCGACACCAAGGTGTATTGCTACTACAACACCGCGCTGGAATCCGAGCG     836
ATCC9027           GCCGGGAGGCATCGATACCAAGGTGTATTGCTACTACAACACCGCGCTGGAGTCCGAGCG     837
ATCC33359          GCCGGGAGGCATCGATACCAAGGTGTATTGCTACTACAACACCGCGCTGGAGTCCGAGCG     830
EML545             GCCGGGAGGCATCGATACCAAGGTGTATTGCTACTACAACACCGCGCTGGAGTCCGAGCG     830
AR_0356            GCCGGGAGGCATCGATACCAAGGTGTATTGCTACTACAACACCGCGCTGGAGTCCGAGCG     821
AR441              GCCGGGAGGCATCGATACCAAGGTGTATTGCTACTACAACACCGCGCTGGAGTCCGAGCG     821
WH-SGI-V-07165     GCCGGGAGGCATCGATACCAAGGTGTATTGCTACTACAACACCGCGCTGGAGTCCGAGCG     821
EML1796            GCCGGGAGGCATCGATACCAAGGTGTATTGCTACTACAACACCGCGCTGGAGTCCGAGCG     821
EML1795            GCCGGGAGGCATCGATACCAAGGTGTATTGCTACTACAACACCGCGCTGGAGTCCGAGCG     821
MIN-137            GCCGGGAGGCATCGATACCAAGGTGTATTGCTACTACAACACCGCGCTGGAGTCCGAGCG     823
PSA00358           GCCGGGAGGCATCGATACCAAGGTGTATTGCTACTACAACACCGCGCTGGAGTCCGAGCG     836
PSA00289           GCCGGGAGGCATCGATACCAAGGTGTATTGCTACTACAACACCGCGCTGGAGTCCGAGCG     836
PA7                GCCGGGTGGCATCGATACCAAGGTGTATTGCTACTACAACACCGCGCTGGAGTCCGAGCG     834
PABL043            GCCGGGTGGCATCGATACCAAGGTGTATTGCTACTACAACACCGCGCTGGAGTCCGAGCG     834
WH-SGI-V-07064     GCCGGGTGGCATCGATACCAAGGTGTATTGCTACTACAACACCGCGCTGGAGTCCGAGCG     834
WH-SGI-V-07072     GCCGGGTGGCATCGATACCAAGGTGTATTGCTACTACAACACCGCGCTGGAGTCCGAGCG     834
MAZ105             GCCGGGTGGCATCGATACCAAGGTGTATTGCTACTACAACACCGCGCTGGAGTCCGAGCG     834
WH-SGI-V-07370     GCCGGGTGGCATCGATACCAAGGTGTATTGCTACTACAACACCGCGCTGGAGTCCGAGCG     839
                    ***** ******** *********************************** ********

PAO1               CAGCTTCCTGCGCTTTCTCGAAAGCGCCCGCCAGCGCCTGCGCGAACTCGGCCGCCAGCG     896
ATCC9027           CAGCTTCCTGCGCTTTCTCGAAAGCGCCCGCCAGCGCCTGCGCGAACTGGGCCGGCAGCG     897
ATCC33359          CAGCTTCCTGCGCTTTCTCGAAAGCGCCCGCCAGCGCCTGCGCGAACTGGGCCGGCAGCG     890
EML545             CAGCTTCCTGCGCTTTCTCGAAAGCGCCCGCCAGCGCCTGCGCGAACTGGGCCGGCAGCG     890
AR_0356            CAGCTTCCTGCGCTTTCTCGAAAGCGCCCGCCAGCGCCTGCGCGAACTGGGCCGGCAGCG     881
AR441              CAGCTTCCTGCGCTTTCTCGAAAGCGCCCGCCAGCGCCTGCGCGAACTGGGCCGGCAGCG     881
WH-SGI-V-07165     CAGCTTCCTGCGCTTTCTCGAAAGCGCCCGCCAGCGCCTGCGCGAACTGGGCCGGCAGCG     881
EML1796            CAGCTTCCTGCGCTTTCTCGAAAGCGCCCGCCAGCGCCTGCGCGAACTGGGCCGGCAGCG     881
EML1795            CAGCTTCCTGCGCTTTCTCGAAAGCGCCCGCCAGCGCCTGCGCGAACTGGGCCGGCAGCG     881
MIN-137            CAGCTTCCTGCGCTTTCTCGAAAGCGCCCGCCAGCGCCTGCGCGAACTGGGCCGGCAGCG     883
PSA00358           CAGCTTCCTGCGCTTTCTCGAAAGCGCCCGCCAGCGCCTGCGCGAACTGGGCCGGCAGCG     896
PSA00289           CAGCTTCCTGCGCTTTCTCGAAAGCGCCCGCCAGCGCCTGCGCGAACTGGGCCGGCAGCG     896
PA7                CAGCTTCCTGCGCTTTCTCGAAAGCGCCCGCCAGCGCCTGCGCGAACTGGGCCGGCAGCG     894
PABL043            CAGCTTCCTGCGCTTTCTCGAAAGCGCCCGCCAGCGCCTGCGCGAACTGGGCCGGCAGCG     894
WH-SGI-V-07064     CAGCTTCCTGCGCTTTCTCGAAAGCGCCCGCCAGCGCCTGCGCGAACTGGGCCGGCAGCG     894
WH-SGI-V-07072     CAGCTTCCTGCGCTTTCTCGAAAGCGCCCGCCAGCGCCTGCGCGAACTGGGCCGGCAGCG     894
MAZ105             CAGCTTCCTGCGCTTTCTCGAAAGCGCCCGCCAGCGCCTGCGCGAACTGGGCCGGCAGCG     894
WH-SGI-V-07370     CAGCTTCCTGCGCTTTCTCGAAAGCGCCCGCCAGCGCCTGCGCGAACTGGGCCGGCAGCG     899
                   ************************************************ ***** *****

PAO1               TTTCGACGATGCGCCGGCCTGGCAACCGAGCATCGTCGAAACGGCGCAGCGCCGCTCAGG     956
ATCC9027           CTTCGACGAGGCGCCGGCCTGGCAGCCGAGCATCGTCGAGACGGTGCCACGGTGCTCAGG     957
ATCC33359          CTTCGACGAGGCGCCGGCCTGGCAGCCGAGCATCGTCGAGACGGTGCCACGGTGCTCAGG     950
EML545             CTTCGACGAGGCGCCGGCCTGGCAGCCGAGCATCGTCGAGACGGTGCCACGGTGCTCAGG     950
AR_0356            CTTCGACGAGGCGCCGGCCTGGCAGCCGAGCATCGTCGAGACGGTGCCACGGTGCTCAGG     941
AR441              CTTCGACGAGGCGCCGGCCTGGCAGCCGAGCATCGTCGAGACGGTGCCACGGTGCTCAGG     941
WH-SGI-V-07165     CTTCGACGAGGCGCCGGCCTGGCAGCCGAGCATCGTCGAGACGGTGCCACGGTGCTCAGG     941
EML1796            CTTCGACGAGGCGCCGGCCTGGCAGCCGAGCATCGTCGAGACGGTGCCACGGTGCTCAGG     941
EML1795            CTTCGACGAGGCGCCGGCCTGGCAGCCGAGCATCGTCGAGACGGTGCCACGGTGCTCAGG     941
MIN-137            CTTCGACGAGGCGCCGGCCTGGCAGCCGAGCATCGTCGAGACGGTGCCACGGTGCTCAGG     943
PSA00358           CTTCGACGAGGCGCCGGCCTGGCAGCCGAGCATCGTCGAGACGGTGCCACGGTGCTCAGG     956
PSA00289           CTTCGACGAGGCGCCGGCCTGGCAGCCGAGCATCGTCGAGACGGTGCCACGGTGCTCAGG     956
PA7                CTTCGACGAGGCGCCGGCCTGGCAGCCGAGCATCGTCGAGACGGTGCCACGGTGCTCAGG     954
PABL043            CTTCGACGAGGCGCCGGCCTGGCAGCCGAGCATCGTCGAGACGGTGCCACGGTGCTCAGG     954
WH-SGI-V-07064     CTTCGACGAGGCGCCGGCCTGGCAGCCGAGCATCGTCGAGACGGTGCCACGGTGCTCAGG     954
```

```
WH-SGI-V-07072      CTTCGACGAGGCGCCGGCCTGGCAGCCGAGCATCGTCGAGACGGTGCCACGGTGCTCAGG    954
MAZ105              CTTCGACGAGGCGCCGGCCTGGCAGCCGAGCATCGTCGAGACGGTGCCACGGTGCTCAGG    954
WH-SGI-V-07370      CTTCGACGAGGCGCCGGCCTGGCAGCCGAGCATCGTCGAGACGGTGCCACGGTGCTCAGG    959
                     ******** ************** ************** **** **  **  *******

PAO1                CCCGAAGGCGCTCGCGTACCGCCAGCGCGCCGCACCAGAGTAG    999
ATCC9027            CCCGAAGGCGCTCGCGTACCGGCAGCGCGCCGCACCAGAGTAG    1000
ATCC33359           CCCGAAGGCGCTCGCGTACCGGCAGCGCGCCGCACCAGAGTAG    993
EML545              CCCGAAGGCGCTCGCGTACCGGCAGCGCGCCGCACCAGAGTAG    993
AR_0356             CCCGAAGGCGCTCGCGTACCGGCAGCGCGCCGCACCAGAGTAG    984
AR441               CCCGAAGGCGCTCGCGTACCGGCAGCGCGCCGCACCAGAGTAG    984
WH-SGI-V-07165      CCCGAAGGCGCTCGCGTACCGGCAGCGCGCCGCACCAGAGTAG    984
EML1796             CCCGAAGGCGCTCGCGTACCGGCAGCGCGCCGCACCAGAGTAG    984
EML1795             CCCGAAGGCGCTCGCGTACCGGCAGCGCGCCGCACCAGAGTAG    984
MIN-137             CCCGAAGGCGCTCGCGTACCGGCAGCGCGCCGCACCAGAGTAG    986
PSA00358            CCCGAAGGCGCTCGCGTACCGGCAGCGCGCCGCACCAGAGTAG    999
PSA00289            CCCGAAGGCGCTCGCGTACCGGCAGCGCGCCGCACCAGAGTAG    999
PA7                 CCCGAAGGCGCTCGCGTACCGGCAGCGCGCCGCACCAGAGTAG    997
PABL043             CCCGAAGGCGCTCGCGTACCGGCAGCGCGCCGCACCAGAGTAG    997
WH-SGI-V-07064      CCCGAAGGCGCTCGCGTACCGGCAGCGCGCCGCACCAGAGTAG    997
WH-SGI-V-07072      CCCGAAGGCGCTCGCGTACCGGCAGCGCGCCGCACCAGAGTAG    997
MAZ105              CCCGAAGGCGCTCGCGTACCGGCAGCGCGCCGCACCAGAGTAG    997
WH-SGI-V-07370      CCCGAAGGCGCTCGCGTACCGGCAGCGCGCCGCACCAGAGTAG    1002
                    ********************* *********************
```

Amino acid sequence alignment of PqsR

```
CLUSTAL O(1.2.4) multiple sequence alignment


MIN-137             MPIHNLNHVNMFLQVIASGSISSAARILRKSHTAVSSAVSNLEIDLCVELVRRDGYKVEP    60
PAO1                MPIHNLNHVNMFLQVIASGSISSAARILRKSHTAVSSAVSNLEIDLCVELVRRDGYKVEP    60
AR441               MPIHNLNHVNMFLQVIASGSISSAARILRKSHTAVSSAVSNLEIDLCVELVRRDGYKVEP    60
AR_0356             MPIHNLNHVNMFLQVIASGSISSAARILRKSHTAVSSAVSNLEIDLCVELVRRDGYKVEP    60
WH-SGI-V-07165      MPIHNLNHVNMFLQVIASGSISSAARILRKSHTAVSSAVSNLEIDLCVELVRRDGYKVEP    60
EML1796             MPIHNLNHVNMFLQVIASGSISSAARILRKSHTAVSSAVSNLEIDLCVELVRRDGYKVEP    60
EML1795             MPIHNLNHVNMFLQVIASGSISSAARILRKSHTAVSSAVSNLEIDLCVELVRRDGYKVEP    60
ATCC_33359          MPIHNLNHVNMFLQVIASGSISSAARILRKSHTAVSSAVSNLEIDLCVELVRRDGYKVEP    60
EML545              MPIHNLNHVNMFLQVIASGSISSAARILRKSHTAVSSAVSNLEIDLCVELVRRDGYKVEP    60
PSA00358            MPIHNLNHVNMFLQVIASGSISSAARILRKSHTAVSSAVSNLEIDLCVELVRRDGYKVEP    60
PSA00289            MPIHNLNHVNMFLQVIASGSISSAARILRKSHTAVSSAVSNLEIDLCVELVRRDGYKVEP    60
MAZ105              MPIHNLNHVNMFLQVIASGSISSAARILRKSHTAVSSAVSNLEIDLCVELVRRDGYKVEP    60
PA7                 MPIHNLNHVNMFLQVIASGSISSAARILRKSHTAVSSAVSNLEIDLCVELVRRDGYKVEP    60
PABL043             MPIHNLNHVNMFLQVIASGSISSAARILRKSHTAVSSAVSNLEIDLCVELVRRDGYKVEP    60
WH-SGI-V-07064      MPIHNLNHVNMFLQVIASGSISSAARILRKSHTAVSSAVSNLEIDLCVELVRRDGYKVEP    60
WH-SGI-V-07072      MPIHNLNHVNMFLQVIASGSISSAARILRKSHTAVSSAVSNLEIDLCVELVRRDGYKVEP    60
ATCC9027            MPIHNLNHVNMFLQVIASGSISSAARILRKSHTAVSSAVSNLKIDLCVELVRRDGYKVEP    60
                    ******************************************:*****************

MIN-137             TEQALRLIPYMRSLLNYQQLIGDIAFNLNKGPRNLRVLLDTAIPPSFCDTVSSVLLDDFN    120
PAO1                TEQALRLIPYMRSLLNYQQLIGDIAFNLNKGPRNLRVLLDTAIPPSFCDTVSSVLLDDFN    120
AR441               TEQALRLIPYMRSLLNYQQLIGDIAFNLNKGPRNLRVLLDTAIPPSFCDTVSSVLLDDFN    120
AR_0356             TEQALRLIPYMRSLLNYQQLIGDIAFNLNKGPRNLRVLLDTAIPPSFCDTVSSVLLDDFN    120
WH-SGI-V-07165      TEQALRLIPYMRSLLNYQQLIGDIAFNLNKGPRNLRVLLDTAIPPSFCDTVSSVLLDDFN    120
EML1796             TEQALRLIPYMRSLLNYQQLIGDIAFNLNKGPRNLRVLLDTAIPPSFCDTVSSVLLDDFN    120
EML1795             TEQALRLIPYMRSLLNYQQLIGDIAFNLNKGPRNLRVLLDTAIPPSFCDTVSSVLLDDFN    120
ATCC_33359          TEQALRLIPYMRSLLNYQQLIGDIAFNLNKGPRNLRVLLDTAIPPSFCDTVSSVLLDDFN    120
EML545              TEQALRLIPYMRSLLNYQQLIGDIAFNLNKGPRNLRVLLDTAIPPSFCDTVSSVLLDDFN    120
PSA00358            TEQALRLIPYMRSLLNYQQLIGDIAFNLNKGPRNLRVLLDTAIPPSFCDTVSSVLLDDFN    120
PSA00289            TEQALRLIPYMRSLLNYQQLIGDIAFNLNKGPRNLRVLLDTAIPPSFCDTVSSVLLDDFN    120
MAZ105              TEQALRLIPYMRSLLNYQQLIGDIAFNLNKGPRNLRVLLDTAIPPSFCDTVSSVLLDDFN    120
PA7                 TEQALRLIPYMRSLLNYQQLIGDIAFNLNKGPRNLRVLLDTAIPPSFCDTVSSVLLDDFN    120
PABL043             TEQALRLIPYMRSLLNYQQLIGDIAFNLNKGPRNLRVLLDTAIPPSFCDTVSSVLLDDFN    120
WH-SGI-V-07064      TEQALRLIPYMRSLLNYQQLIGDIAFNLNKGPRNLRVLLDTAIPPSFCDTVSSVLLDDFN    120
WH-SGI-V-07072      TEQALRLIPYMRSLLNYQQLIGDIAFNLNKGPRNLRVLLDTAIPPSFCDTVSSVLLDDFN    120
```

```
ATCC9027           TEQALRLIPYMRSLLNYQQLIGDIAFNLNKGPRNLRVLLDTAIPPSFCDTVSSVLLDDFN   120
                   ************************************************************

MIN-137            MVSLIRTSPADSLATIKQDNAEIDIAITIDEELKISRFNQCVLGYTKAFVVAHPQHPLCN   180
PAO1               MVSLIRTSPADSLATIKQDNAEIDIAITIDEELKISRFNQCVLGYTKAFVVAHPQHPLCN   180
AR441              MVSLIRTSPADSLATIKQDNAEIDIAITI-----ISRFNQCVLGYTKAFVVAHPQHPLCN   175
AR_0356            MVSLIRTSPADSLATIKQDNAEIDIAITI-----ISRFNQCVLGYTKAFVVAHPQHPLCN   175
WH-SGI-V-07165     MVSLIRTSPADSLATIKQDNAEIDIAITI-----ISRFNQCVLGYTKAFVVAHPQHPLCN   175
EML1796            MVSLIRTSPADSLATIKQDNAEIDIAITI-----ISRFNQCVLGYTKAFVVAHPQHPLCN   175
EML1795            MVSLIRTSPADSLATIKQDNAEIDIAITI-----ISRFNQCVLGYTKAFVVAHPQHPLCN   175
ATCC_33359         MVSLIRTSPADSLATIKQDNAEIDIA--IDEELKISRFNQCVLGYTKAFVVAHPQHPLCN   178
EML545             MVSLIRTSPADSLATIKQDNAEIDIA--IDEELKISRFNQCVLGYTKAFVVAHPQHPLCN   178
PSA00358           MVSLIRTSPADSLATIK-------------------------------------------   137
PSA00289           MVSLIRTSPADSLATIK-------------------------------------------   137
MAZ105             MVSLIRTSPADSLATIKQDNAEIDIAITIDEELKISRFNQCVLGYTKAFVVAHPQHPLCN   180
PA7                MVSLIRTSPADSLATIKQDNAEIDIAITIDEELKISRFNQCVLGYTKAFVVAHPQHPLCN   180
PABL043            MVSLIRTSPADSLATIKQDNAEIDIAITIDEELKISRFNQCVLGYTKAFVVAHPQHPLCN   180
WH-SGI-V-07064     MVSLIRTSPADSLATIKQDNAEIDIAITIDEELKISRFNQCVLGYTKAFVVAHPQHPLCN   180
WH-SGI-V-07072     MVSLIRTSPADSLATIKQDNAEIDIAITIDEELKISRFNQCVLGYTKAFVVAHPQHPLCN   180
ATCC9027           MVSLIRTSPADSLATIKQDNAEIDIAITIDEELKISRFNQCVLGYTKAFVVAHPQHPLCN   180
                   ***************** ********  *     **************************

MIN-137            ASLHSIASLANYRQISLGSRFGQHSNLLRPVSDKVLFVENFDDMLRLVEAGVGRTISSRS   240
PAO1               ASLHSIASLANYRQISLGSRSGQHSNLLRPVSDKVLFVENFDDMLRLVEAGVGWGIAPHY   240
AR441              ASLHSIASLANYRQISLGSRFGQHSNLLRPVSDKVLFVENFDDMLRLVEAGVGWGIAPHY   235
AR_0356            ASLHSIASLANYRQISLGSRFGQHSNLLRPVSDKVLFVENFDDMLRLVEAGVGWGIAPHY   235
WH-SGI-V-07165     ASLHSIASLANYRQISLGSRFGQHSNLLRPVSDKVLFVENFDDMLRLVEAGVGWGIAPHY   235
EML1796            ASLHSIASLANYRQISLGSRFGQHSNLLRPVSDKVLFVENFDDMLRLVEAGVGWGIAPHY   235
EML1795            ASLHSIASLANYRQISLGSRFGQHSNLLRPVSDKVLFVENFDDMLRLVEAGVGWGIAPHY   235
ATCC_33359         ASLHSIASLANYRQISLGSRFGQHSNLLRPVSDKVLFVENFDDMLRLVEAGVGWGIAPHY   238
EML545             ASLHSIASLANYRQISLGSRFGQHSNLLRPVSDKVLFVENFDDMLRLVEAGVGWGIAPHY   238
PSA00358           ------------------------------------------------------------   137
PSA00289           ------------------------------------------------------------   137
MAZ105             ASLHSIASLANYRQISLGSRFGQHSNLLRPVSDKVLFVENFDDMLRLVEAGVV-------   239
PA7                ASLHSIASLANYRQISLGSRFGQHSNLLRPVSDKVLFVENFDDMLRLVEAGVV-GYRAAL   239
PABL043            ASLHSIASLANYRQISLGSRFGQHSNLLRPVSDKVLFVENFDDMLRLVEAGVV-GYRAAL   239
WH-SGI-V-07064     ASLHSIASLANYRQISLGSRFGQHSNLLRPVSDKVLFVENFDDMLRLVEAGVV-GYRAAL   239
WH-SGI-V-07072     ASLHSIASLANYRQISLGSRFGQHSNLLRPVSDKVLFVENFDDMLRLVEAGVV-GYRAAL   239
ATCC9027           ASLHSIASLANYRQISLGSRFGQHSNLLRPVSDKVLFVENFDDMLRLVEAGVGWGIAPHY   240
                   ******************** *******************************

MIN-137            ACAPVPWP-----------------------------------FSASCTSREASIPRCIA   265
PAO1               FVEERLRNGTLAVLSELYEPGGIDTKVYCYYNTALESERSFLRFLESARQRLRELGRQRF   300
AR441              FVEERLRAGTLAVLSELYEPGGIDTKVYCYYNTALESERSFLRFLESARQRLRELGRQRF   295
AR_0356            FVEERLRAGTLAVLSELYEPGGIDTKVYCYYNTALESERSFLRFLESARQRLRELGRQRF   295
WH-SGI-V-07165     FVEERLRAGTLAVLSELYEPGGIDTKVYCYYNTALESERSFLRFLESARQRLRELGRQRF   295
EML1796            FVEERLRAGTLAVLSELYEPGGIDTKVYCYYNTALESERSFLRFLESARQRLRELGRQRF   295
EML1795            FVEERLRAGTLAVLSELYEPGGIDTKVYCYYNTALESERSFLRFLESARQRLRELGRQRF   295
ATCC_33359         FVEERLRAGTLAVLSELYEPGGIDTKVYCYYNTALESERSFLRFLESARQRLRELGRQRF   298
EML545             FVEERLRAGTLAVLSELYEPGGIDTKVYCYYNTALESERSFLRFLESARQRLRELGRQRF   298
PSA00358           ------------------------------------------------------------   137
PSA00289           ------------------------------------------------------------   137
MAZ105             FRRGAPTHRHPGRSQRAVRAGWHR-----YQGVLLL------QHRAGVRAQLPALSRKRP   288
PA7                FRRGAPTHRHPGRSQRAVRAGWHR-----YQGVLLL------QHRAGVRAQLPALSRKRP   288
PABL043            FRRGAPTHRHPGRSQRAVRAGWHR-----YQGVLLL------QHRAGVRAQLPALSRKRP   288
WH-SGI-V-07064     FRRGAPTHRHPGRSQRAVRAGWHR-----YQGVLLL------QHRAGVRAQLPALSRKRP   288
WH-SGI-V-07072     FRRGAPTHRHPGRSQRAVRAGWHR-----YQGVLLL------QHRAGVRAQLPALSRKRP   288
ATCC9027           FVEERLRAGTLGRSQRAVRAGRHR-----YQGVLLL------QHRAGVRAQLPALSRKRP   289
                                                              .  .   :   : *
```

```
MIN-137             TTTPRWSPSAASCAFSKAPASACANWAGSASTRRRPGSRASSRRCHGAQARRRSRTGSAP     325
PAO1                DDAPAWQPSIVETAQRRSGPKALAYRQRAAPE----------------------------     332
AR441               DEAPAWQPSIVETVPRCSGPKALAYRQRAAPE----------------------------     327
AR_0356             DEAPAWQPSIVETVPRCSGPKALAYRQRAAPE----------------------------     327
WH-SGI-V-07165      DEAPAWQPSIVETVPRCSGPKALAYRQRAAPE----------------------------     327
EML1796             DEAPAWQPSIVETVPRCSGPKALAYRQRAAPE----------------------------     327
EML1795             DEAPAWQPSIVETVPRCSGPKALAYRQRAAPE----------------------------     327
ATCC_33359          DEAPAWQPSIVETVPRCSGPKALAYRQRAAPE----------------------------     330
EML545              DEAPAWQPSIVETVPRCSGPKALAYRQRAAPE----------------------------     330
PSA00358            ------------------------------------------------------------     137
PSA00289            ------------------------------------------------------------     137
MAZ105              P------------APARTGPAALRRGAGLAAEHRRDGATVLRPEGARVPAARRTRV----     332
PA7                 P------------APARTGPAALRRGAGLAAEHRRDGATVLRPEGARVPAARRTRV----     332
PABL043             P------------APARTGPAALRRGAGLAAEHRRDGATVLRPEGARVPAARRTRV----     332
WH-SGI-V-07064      P------------APARTGPAALRRGAGLAAEHRRDGATVLRPEGARVPAARRTRV----     332
WH-SGI-V-07072      P------------APARTGPAALRRGAGLAAEHRRDGATVLRPEGARVPAARRTRV----     332
ATCC9027            P------------APARTGPAALRRGAGLAAEHRRDGATVLRPEGARVPAARRTRV----     333
                                 .   :   *       *

MIN-137             HQS   328
PAO1                ---   332
AR441               ---   327
AR_0356             ---   327
WH-SGI-V-07165      ---   327
EML1796             ---   327
EML1795             ---   327
ATCC_33359          ---   330
EML545              ---   330
PSA00358            ---   331
PSA00289            ---   331
MAZ105              ---   332
PA7                 ---   332
PABL043             ---   332
WH-SGI-V-07064      ---   332
WH-SGI-V-07072      ---   332
ATCC9027            ---   333
```

Nucleotide sequence alignment of *lasR*

```
CLUSTAL O(1.2.4) multiple sequence alignment


PAO1                    ATGGCCTTGGTTGACGGTTTTCTTGAGCTGGAACGCTCAAGTGGAAAATTGGAGTGGAGC      60
MAZ105                  ATGGCCTTGGTTGACGGTTTTCTTGAGCTGGAACGCTCAAGTGGGAAATTGGAATGGAGC      60
WH-SGI-V-07064          ATGGCCTTGGTTGACGGTTTTCTTGAGCTGGAACGCTCAAGTGGGAAATTGGAATGGAGC      60
515477                  ATGGCCTTGGTTGACGGTTTTCTTGAGCTGGAACGCTCAAGTGGGAAATTGGAATGGAGC      60
AZPAE14941              ATGGCCTTGGTTGACGGTTTTCTTGAGCTGGAACGCTCAAGTGGGAAATTGGAATGGAGC      60
LMG5031                 ATGGCCTTGGTTGACGGTTTTCTTGAGCTGGAACGCTCAAGTGGGAAATTGGAATGGAGC      60
WH-SGI-V-07287          ATGGCCTTGGTTGACGGTTTTCTTGAGCTGGAACGCTCAAGTGGGAAATTGGAATGGAGC      60
PA7                     ATGGCCTTGGTTGACGGTTTTCTTGAGCTGGAACGCTCAAGTGGGAAATTGGAATGGAGC      60
EML528                  ATGGCCTTGGTTGACGGTTTTCTTGAGCTGGAACGCTCAAGTGGGAAATTGGAATGGAGC      60
                        ******************************************** ******** ******

PAO1                    GCCATCCTGCAGAAGATGGCGAGCGACCTTGGATTCTCGAAGATCCTGTTCGGCCTGTTG      120
MAZ105                  GCCATCCTGCAGAAGATGGCGAGCGACCTGGGGTTTTCGAAAATCCTGTTCGGCCTGCTA      120
WH-SGI-V-07064          GCCATCCTGCAGAAGATGGCGAGCGACCTGGGGTTTTCGAAAATCCTGTTCGGCCTGCTA      120
515477                  GCCATCCTGCAGAAGATGGCGAGCGACCTGGGGTTTTCGAAAATCCTGTTCGGCCTGCTA      120
AZPAE14941              GCCATCCTGCAGAAGATGGCGAGCGACCTGGGGTTTTCGAAAATCCTGTTCGGCCTGCTA      120
LMG5031                 GCCATCCTGCAGAAGATGGCGAGCGACCTGGGGTTTTCGAAAATCCTGTTCGGCCTGCTA      120
WH-SGI-V-07287          GCCATCCTGCAGAAGATGGCGAGCGACCTGGGGTTTTCGAAAATCCTGTTCGGCCTGCTA      120
PA7                     GCCATCCTGCAGAAGATGGCGAGCGACCTGGGGTTTTCGAAAATCCTGTTCGGCCTGCTA      120
EML528                  GCCATCCTGCAGAAGATGGCGAGCGACCTGGGGTTTTCGAAAATCCTGTTCGGCCTGCTA      120
                        ***************************** ** ** ***** *************** * 

PAO1                    CCTAAGGACAGCCAGGACTACGAGAACGCCTTCATCGTCGGCAACTACCCGGCCGCCTGG      180
MAZ105                  CCGAAGGACAGCCAGGACTACGAGAACGCCTTCATCGTCGGCAACTACCCGGCCGCCTGG      180
WH-SGI-V-07064          CCGAAGGACAGCCAGGACTACGAGAACGCCTTCATCGTCGGCAACTACCCGGCCGCCTGG      180
515477                  CCGAAGGACAGCCAGGACTACGAGAACGCCTTCATCGTCGGCAACTACCCGGCCGCCTGG      180
AZPAE14941              CCGAAGGACAGCCAGGACTACGAGAACGCCTTCATCGTCGGCAACTACCCGGCCGCCTGG      180
LMG5031                 CCGAAGGACAGCCAGGACTACGAGAACGCCTTCATCGTCGGCAACTACCCGGCCGCCTGG      180
WH-SGI-V-07287          CCGAAGGACAGCCAGGACTACGAGAACGCCTTCATCGTCGGCAACTACCCGGCCGCCTGG      180
PA7                     CCGAAGGACAGCCAGGACTACGAGAACGCCTTCATCGTCGGCAACTACCCGGCCGCCTGG      180
EML528                  CCGAAGGACAGCCAGGACTACGAGAACGCCTTCATCGTCGGCAACTACCCGGCCGCCTGG      180
                        ** *********************************************************

PAO1                    CGCGAGCATTACGACCGGGCTGGCTACGCGCGGGTCGACCCGACGGTCAGTCACTGTACC      240
MAZ105                  CGCGAGCATTACGACAAGGCCGGCTATGCGCGGGTCGACCCGACGGTCAGCCATTGTACC      240
WH-SGI-V-07064          CGCGAGCATTACGACAAGGCCGGCTATGCGCGGGTCGACCCGACGGTCAGCCATTGTACC      240
515477                  CGCGAGCATTACGACAAGGCCGGCTATGCGCGGGTCGACCCGACGGTCAGCCATTGTACC      240
AZPAE14941              CGCGAGCATTACGACAAGGCCGGCTATGCGCGGGTCGACCCGACGGTCAGCCATTGTACC      240
LMG5031                 CGCGAGCATTACGACAAGGCCGGCTATGCGCGGGTCGACCCGACGGTCAGCCATTGTACC      240
WH-SGI-V-07287          CGCGAGCATTACGACAAGGCCGGCTATGCGCGGGTCGACCCGACGGTCAGCCATTGTACC      240
PA7                     CGCGAGCATTACGACAAGGCCGGCTATGCGCGGGTCGACCCGACGGTCAGCCATTGTACC      240
EML528                  CGCGAGCATTACGACAAGGCCGGCTATGCGCGGGTCGACCCGACGGTCAGCCATTGTACC      240
                        ***************  *** ***** *********************** ** ******

PAO1                    CAGAGCGTACTGCCGATTTTCTGGGAACCGTCCATCTACCAGACGCGAAAGCAGCACGAG      300
MAZ105                  CAGAGCGTCCTGCCGATCTTCTGGGAACCCTCCATCTACCAGACACGCAAGCAGCACGAG      300
WH-SGI-V-07064          CAGAGCGTCCTGCCGATCTTCTGGGAACCCTCCATCTACCAGACACGCAAGCAGCACGAG      300
515477                  CAGAGCGTCCTGCCGATCTTCTGGGAACCCTCCATCTGC-------------AGCACGAG      287
AZPAE14941              CAGAGCGTCCTGCCGATCTTCTGGGAACCCTCCATCTACCAGACACGCAAGCAGCACGAG      300
LMG5031                 CAGAGCGTCCTGCCGATCTTCTGGGAACCCTCCATCTACCAGACACGCAAGTAGCACGAG      300
WH-SGI-V-07287          CAGAGCGTCCTGCCGATCTTCTGGGAACCCTCCATCTACCAGACACGCAAGTAGCACGAG      300
PA7                     CAGAGCGTCCTGCCGATCTTCTGGGAACCCTCCATCTACCAGACACGCAAGCAGCACGAG      300
EML528                  CAGAGCGTCCTGCCGATCTTCTAGGAACCCTCCATCTACCAGACACGCAAGCAGCACGAG      300
                        ******** ******** **** ****** ******* *             ********

PAO1                    TTCTTCGAGGAAGCCTCGGCCGCCGGCCTGGTGTATGGGCTGACCATGCCGCTGCATGGT      360
MAZ105                  TTCTTCGAGGAAGCCTCGGCCGCCGGCCTGGTGTACGGGCTGACCATGCCGTTGCACGGT      360
WH-SGI-V-07064          TTCTTCGAGGAAGCCTCGGCCGCCGGCCTGGTGTACGGGCTGACCATGCCGTTGCACGGT      360
515477                  TTCTTCGAGGAAGCCTCGGCCGCCGGCCTGGTGTACGGGCTGACCATGCCGTTGCACGGT      347
AZPAE14941              TTCTTCGAGGAAGCCTCGGCCGCCGGCCTGGTGTACGGGCTGACCATGCCGTTGCACGGT      360
LMG5031                 TTCTTCGAGGAAGCCTCGGCCGCCGGCCTGGTGTACGGGCTGACCATGCCGTTGCACGGT      360
WH-SGI-V-07287          TTCTTCGAGGAAGCCTCGGCCGCCGGCCTGGTGTACGGGCTGACCATGCCGTTGCACGGT      360
PA7                     TTCTTCGAGGAAGCCTCGGCCGCCGGCCTGGTGTACGGGCTGACCATGCCGTTGCACGGT      360
EML528                  TTCTTCGAGGAAGCCTCGGCCGCCGGCCTGGTGTACGGGCTGACCATGCCGTTGCACGGT      360
                        *********************************** *************** **** ***
```

```
PAO1               GCTCGCGGCGAACTCGGCGCGCTGAGCCTCAGCGTGGAAGCGGAAAACCGGGCCGAGGCC      420
MAZ105             GCGCGCGGCGAACTCGGCGCCCTGAGTCTGAGCGTGGAGGCGGAGAGCCGGGTCGAGGCG      420
WH-SGI-V-07064     GCGCGCGGCGAACTCGGCGCCCTGAGTCTGAGCGTGGAGGCGGAGAGCCGGGCCGAGGCG      420
515477             GCGCGCGGCGAACTCGGCGCCCTGAGTCTGAGCGTGGAGGCGGAGAGCCGGGCCGAGGCG      407
AZPAE14941         GCGCGCGGCGAACTCGGCGCCCTGAGTCTGG------------AGAGCCGGGCCGAGGCG      408
LMG5031            GCGCGCGGCGAACTCGGCGCCCTGAGTCTGAGCGTGGAGGCGGAGAGCCGGGGCGAGGCG      420
WH-SGI-V-07287     GCGCGCGGCGAACTCGGCGCCCTGAGTCTGAGCGTGGAGGCGGAGAGCCGGGGCGAGGCG      420
PA7                GCGCGCGGCGAACTCGGCGCCCTGAGTCTGAGCGTGGAGGCGGAGAGCCGGGCCGAGGCG      420
EML528             GCGCGCGGCGAACTCGGCGCCCTGAGTCTGAGCGTGGAGGCGGAGAGCCGGGCCGAGGCG      420
                   ** ***************** ***** **              * * ***** ******

PAO1               AACCGTTTCATGGAGTCGGTCCTGCCGACCCTGTGGATGCTCAAGGACTACGCACTGCAG      480
MAZ105             AATCGCTTCATGGAGTCGGTGCTG--GACCCTTTGGATGCTCAAGGACTACGCACTGCAA      478
WH-SGI-V-07064     AATCGCTTCATGGAGTCGGTGCTGCCGACCCTTTGGATGCTCAAGGACTACGCACTGCAA      480
515477             AATCGCTTCATGGAGTCGGTGCTGCCGACCCTTTGGATGCTCAAGGACTACGCACTGCAA      467
AZPAE14941         AATCGCTTCATGGAGTCGGTGCTGCCGACCCTTTGGATGCTCAAGGACTACGCACTGCAA      468
LMG5031            AATCGCTTCATGGAGTCGGTGCTGCCGACCCTTTGGATGCTCAAGGACTACGCACTGCAA      480
WH-SGI-V-07287     AATCGCTTCATGGAGTCGGTGCTGCCGACCCTTTGGATGCTCAAGGACTACGCACTGCAA      480
PA7                AATCGCTTCATGGAGTCGGTGCTGCCGACCCTTTGGATGCTCAAGGACTACGCACTGCAA      480
EML528             AATCGCTTCATGGAGTCGGTGCTGCCGACCCTTTGGATGCTCAAGGACTACGCACTGCAA      480
                   ** ** ************** ***  ****** **************************

PAO1               AGCGGTGCCGGACTGGCCTTCGAACATCCGGTCAGCAAACCGGTGGTTCTGACCAGCCGG      540
MAZ105             AGCGGCGCCGGACTGGCCTTCGAACATCCGGTGAACAAGCCGGTGGTATTGACCAGCCGG      538
WH-SGI-V-07064     AGCGGCGCCGGAGCTG--------------------------------------------      496
515477             AGCGGCGCCGGACTGGCCTTCGAACATCCGGTGAACAAGCCGGTGGTATTGACCAGCCGG      527
AZPAE14941         AGCGGCGCCGGACTGGCCTTCGAACATCCGGTGAACAAGCCGGTGGTATTGACCAGCCGG      528
LMG5031            AGCGGCGCCGGACTGGCCTTCGAACATCCGGTGAACAAGCCGGTGGTATTGACCAGCCGG      540
WH-SGI-V-07287     AGCGGCGCCGGACTGGCCTTCGAACATCCGGTGAACAAGCCGGTGGTATTGACCAGCCGG      540
PA7                AGCGGCGCCGGACTGGCCTTCGAACATCCGGTGAACAAGCCGGTGGTATTGACCAGCCGG      540
EML528             AGCGGCGCCGGACTGGCCTTCGAACATCCGGTGAACAAGCCGGTGGTATTGACCAGCCGG      540
                   ***** ******   *

PAO1               GAGAAGGAAGTGTTGCAGTGGTGCGCCATCGGCAAGACCAGTTGGGAGATATCGGTTATC      600
MAZ105             GAGAAGGAAGTCCTGCAGTGGTGCGCCATCGGCAAGACCAGTTGGGAGATATCGGTCATC      598
WH-SGI-V-07064     ------------------------------------------------------------      496
515477             GAGAAGGAAGTCCTGCAGTGGTGCGCCATCGGCAAGACCAGTTGGGAGATATCGGTCATC      587
AZPAE14941         GAGAAGGAAGTCCTGCAGTGGTGCGCCATCGGCAAGACCAGTTGGGAGATATCGGTCATC      588
LMG5031            GAGAAGGAAGTCCTGCAGTGGTGCGCCATCGGCAAGACCAGTTGGGAGATATCGGTCATC      600
WH-SGI-V-07287     GAGAAGGAAGTCCTGCAGTGGTGCGCCATCGGCAAGACCAGTTGGGAGATATCGGTCATC      600
PA7                GAGAAGGAAGTCCTGCAGTGGTGCGCCATCGGCAAGACCAGTTGGGAGATATCGGTCATC      600
EML528             GAGAAGGAAGTCCTGCAGTGGTGCGCCATCGGCAAGACCAGTTGGGAGATATCGGTCATC      600

PAO1               TGCAACTGCTCGGAAGCCAATGTGAACTTCCATATGGGAAATATTCGGCGGAAGTTCGGT      660
MAZ105             TGCAACTGCTCGGAAGCCAACGTGAATTTCCATATGGGAAATATCCGGCGGAAGTTTGGT      658
WH-SGI-V-07064     ------------------------------------------------------------      496
515477             TGCAACTGCTCGGAAGCCAACGTGAATTTCCATATGGGAAATATCCGGCGGAAGTTTGGT      647
AZPAE14941         TGCAACTGCTCGGAAGCCAACGTGAATTTCCATATGGGAAATATCCGGCGGAAGTTTGGT      648
LMG5031            TGCAACTGCTCGGAAGCCAACGTGAATTTCCATATGGGAAATATCCGGCGGAAGTTTGGT      660
WH-SGI-V-07287     TGCAACTGCTCGGAAGCCAACGTGAATTTCCATATGGGAAATATCCGGCGGAAGTTTGGT      660
PA7                TGCAACTGCTCGGAAGCCAACGTGAATTTCCATATGGGAAATATCCGGCGGAAGTTTGGT      660
EML528             TGCAACTGCTCGGAAGCCAACGTGAATTTCCATATGGGAAATATCCGGCGGAAGTTTGGT      660

PAO1               GTGACCTCCCGCCGCGTAGCGGCCATTATGGCCGTTAATTTGGGTCTTATTACTCTCTGA      720
MAZ105             GTCACCTCCCGCCGTGTGGCTGCCATTATGGCTGTTAATTTGGGTCTTATTACTCTCTGA      718
WH-SGI-V-07064     ------------------------------------------------------------      496
515477             GTCACCTCCCGCCGTGTGGCTGCCATTATGGCTGTTAATTTGGGTCTTATTACTCTCTGA      707
AZPAE14941         GTCACCTCCCGCCGTGTGGCTGCCATTATGGCTGTTAATTTGGGTCTTATTACTCTCTGA      708
LMG5031            GTCACCTCCCGCCGTGTGGCTGCCATTATGGCTGTTAATTTGGGTCTTATTACTCTCTGA      720
WH-SGI-V-07287     GTCACCTCCCGCCGTGTGGCTGCCATTATGGCTGTTAATTTGGGTCTTATTACTCTCTGA      720
PA7                GTCACCTCCCGCCGTGTGGCTGCCATTATGGCTGTTAATTTGGGTCTTATTACTCTCTGA      720
EML528             GTCACCTCCCGCCGTGTGGCTGCCATTATGGCTGTTAATTTGGGTCTTATTACTCTCTGA      720
```

Amino acid sequence alignment of LasR

```
CLUSTAL O(1.2.4) multiple sequence alignment

PAO1                    MALVDGFLELERSSGKLEWSAILQKMASDLGFSKILFGLLPKDSQDYENAFIVGNYPAAW      60
515477                  MALVDGFLELERSSGKLEWSAILQKMASDLGFSKILFGLLPKDSQDYENAFIVGNYPAAW      60
MAZ105                  MALVDGFLELERSSGKLEWSAILQKMASDLGFSKILFGLLPKDSQDYENAFIVGNYPAAW      60
AZPAE14941              MALVDGFLELERSSGKLEWSAILQKMASDLGFSKILFGLLPKDSQDYENAFIVGNYPAAW      60
LMG5031                 MALVDGFLELERSSGKLEWSAILQKMASDLGFSKILFGLLPKDSQDYENAFIVGNYPAAW      60
WH-SGI-V-07287          MALVDGFLELERSSGKLEWSAILQKMASDLGFSKILFGLLPKDSQDYENAFIVGNYPAAW      60
WH-SGI-V-07064          MALVDGFLELERSSGKLEWSAILQKMASDLGFSKILFGLLPKDSQDYENAFIVGNYPAAW      60
PA7                     MALVDGFLELERSSGKLEWSAILQKMASDLGFSKILFGLLPKDSQDYENAFIVGNYPAAW      60
EML528                  MALVDGFLELERSSGKLEWSAILQKMASDLGFSKILFGLLPKDSQDYENAFIVGNYPAAW      60
                        ************************************************************

PAO1                    REHYDRAGYARVDPTVSHCTQSVLPIFWEPSIYQTRKQHEFFEEASAAGLVYGLTMPLHG      120
515477                  REHYDKAGYARVDPTVSHCTQSVLPIFWEPSICSTSSSRKPRPPAWCTG-PCRCTVRAAN      119
MAZ105                  REHYDKAGYARVDPTVSHCTQSVLPIFWEPSIYQTRKQHEFFEEASAAGLVYGLTMPLHG      120
AZPAE14941              REHYDKAGYARVDPTVSHCTQSVLPIFWEPSIYQTRKQHEFFEEASAAGLVYGLTMPLHG      120
LMG5031                 REHYDKAGYARVDPTVSHCTQSVLPIFWEPSIYQTRK-----------------------       96
WH-SGI-V-07287          REHYDKAGYARVDPTVSHCTQSVLPIFWEPSIYQTRK-----------------------       96
WH-SGI-V-07064          REHYDKAGYARVDPTVSHCTQSVLPIFWEPSIYQTRKQHEFFEEASAAGLVYGLTMPLHG      120
PA7                     REHYDKAGYARVDPTVSHCTQSVLPIFWEPSIYQTRKQHEFFEEASAAGLVYGLTMPLHG      120
EML528                  REHYDKAGYARVDPTVSHCTQSVLPIF---------------------------------       86
                        *****:********************* **** .* . ::    * .:*     *:   .

PAO1                    ARGELGALSLSVEAE-----NR----AEANRFMESVLPTLWMLKDYAL-Q--SGAGLA--      166
515477                  SAP-V-AWRRRAGPRRIASWSRCCRPFGCSRTTHCKAAPDWPSNIR-TSRWY-PAGRRKS      175
MAZ105                  ARGELGALSLSVEAE-----SR----VEANRFMESVLDPLDAQGLRTAKR--RRTGLRT-      168
AZPAE14941              ARGELGALSL----E-----SR----AEANRFMESVLPTLWMLKDYAL-Q--SGAGLA--      162
LMG5031                 ------------------------------------------------------------       96
WH-SGI-V-07287          ------------------------------------------------------------       96
WH-SGI-V-07064          ARGELGALSLSVEAE-----SR----AEANRFMESVLPTLWMLKDYAL-Q--SGAGA---      165
PA7                     ARGELGALSLSVEAE-----SR----AEANRFMESVLPTLWMLKDYAL-Q--SGAGLA--      166
EML528                  ------------------------------------------------------------       86
                        :   : *       .     .*      ..*   ..              :    :*

PAO1                    ------------FEHP--VSKPVVLTSREKEV--------------LQWCAIGKTSWEIS      198
515477                  CSGAPSARPVGRYRSSATARKPT-ISIWEISGGS-----LVS--PPAVWLPLWLLIWVLL      227
MAZ105                  -----SGEQAGGIDQPG-EGSPAVVRHRQDQLGDIGHLQLLGSQREFPYGKYPAEVWCHL      222
AZPAE14941              ------------FEHP--VNKPVVLTSREKEV--------------LQWCAIGKTSWEIS      194
LMG5031                 ------------------------------------------------------------       96
WH-SGI-V-07287          ------------------------------------------------------------       96
WH-SGI-V-07064          ------------------------------------------------------------      165
PA7                     ------------FEHP--VNKPVVLTSREKEV--------------LQWCAIGKTSWEIS      198
EML528                  ------------------------------------------------------------       86

PAO1                    VICNCSEANVNFHMGNIRRKFGVTSRRVAAIMAVNLGLITL      239
515477                  LS---------------------------------------      229
MAZ105                  PPCGCHYGC----FGSY---YSL------------------      238
AZPAE14941              VICNCSEANVNFHMGNIRRKFGVTSRRVAAIMAVNLGLITL      235
LMG5031                 -----------------------------------------       96
WH-SGI-V-07287          -----------------------------------------       96
WH-SGI-V-07064          -----------------------------------------      165
PA7                     VICNCSEANVNFHMGNIRRKFGVTSRRVAAIMAVNLGLITL      239
EML528                  -----------------------------------------       86
```

Figure S3. PAO1 PqsR containing S201F is able to complement the PAO1 Δ*pqsR* mutant for PYO production.

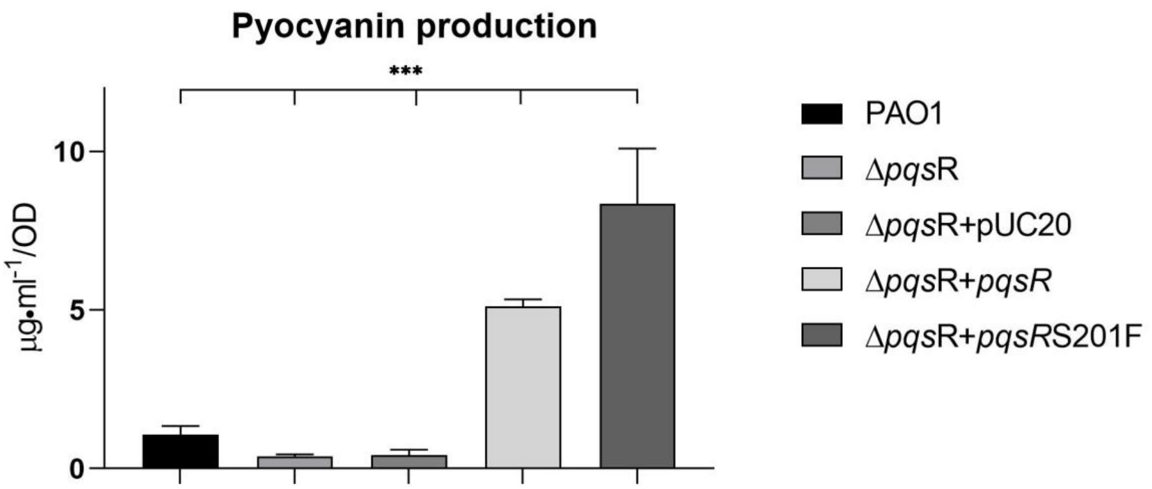

Table S1. List of the 47 clade 3 strains bioinformaticaly analyzed in this work.

Origin of the clade 3 strains		
**Name**	**Isolation source**	**Country**
PA7	Clinical isolation	Argentina
ATCC9027	Clinical isolation	Australia
NCTC13628	Environmental isolation	United Kingdom
AZPAE14901	Clinical isolation	India
EML548	Clinical isolation	Germany
ATCC33359	Environmental isolation	Denmark
EML545	Clinical isolation	Germany
WH-SGI-V-07370	Clinical isolation	France
EML528	Clinical isolation	Germany
AZPAE14941	Clinical isolation	China
CLJ3	Clinical isolation	France
MRSN3705	Clinical isolation	USA
CLJ1	Clinical isolation	France
AUS483	Clinical isolation	Australia
CR1	Environmental isolation	India
PABL043	Clinical isolation	USA
WH-SGI-V-07064	Clinical isolation	USA
WH-SGI-V-07072	Clinical isolation	USA
WH-SGI-V-07055	Clinical isolation	USA
WH-SGI-V-07618	Clinical isolation	USA
515477	Unknown	USA
VRFPA01	Clinical isolation	India
MRSN8141	Clinical isolation	USA
MRSN6241	Clinical isolation	USA
LMG5031	Environmental isolation	Puerto Rico
WH-SGI-V-07234	Environmental isolation	USA
WH-SGI-V-07261	Clinical isolation	Belgium
AZPAE15042	Clinical isolation	Germany
paerg007	Clinical isolation	Switzerland
AUS217	Environmental isolation	Australia
AR_0356	Clinical isolation	Unknown
AR441	Clinical isolation	Unknown
WH-SGI-V-07287	Environmental isolation	Puerto Rico
WH-SGI-V-07165	Clinical isolation	France
KCJ3K67	Clinical isolation	USA
EML1793	Environmental isolation	France
MIN-137	Clinical isolation	United Kingdom
A39-1	Environmental isolation	China
PSA00043	Clinical isolation	USA
PSA00289	Clinical isolation	USA
PSA00304	Clinical isolation	USA
PSA00358	Clinical isolation	USA
EML1796	Environmental isolation	France
EML1795	Environmental isolation	France
PSA00066	Clinical isolation	USA
PSA00320	Clinical isolation	USA
MAZ105	Environmental isolation	Mexico

Table S2. Strains and plasmids used in this work

Bacterial strain	Genotype/Description	Reference
***Pseudomonas aeruginosa* strains**		
MAZ105	Wild type strain isolated from tomato rhizosphere, Mexico	This study
PAO1	Wild type strain isolated in Australia, in 1950, from an infected wound	2
PAO1ΔpqsR	ΔpqsR::Apra^R-FRT	3
PAO1 *pqsA::lux*	CTX::*PpqsA´luxCDABE* tet^R	4
UCBPP-PA14 (PA14)	Wild type strain isolated from a burn patient in USA, 2005.	5
***Escheria coli* strains**		
Dh5α	φ80Δ*lacZ*ΔM15Δ[*lacZYAargF*]U169 *endA recA1 hsdR17 deoR thi-1 supE44*	Invitrogen
**Plasmids**		
pUCP20	Expression vector with *plac* promoter able to replicate in *P. aeruginosa* and *E. coli*; Cb^R	6
pUC20-pqsR	p*lac-pqsR* from PAO1 cloned in pUCP20 Cb^R	21
pUC20-pqsRS201F	Mutation puntual on *pqsR*S201F	This study
pUC20-pqsABCDE	p*lac-pqsABCDE* from PAO1 cloned in pUCP20 Cb^R	This study
pUCP20-pqsE	p*lac-pqsE* from PAO1 cloned in pUCP20 Cb^R	21

Table S3. Oligonucleotides used in this work

Name	Oligonucleotide sequence 5' – 3'
PqsRS201Fa*	CAGCCTCGGCAGCCGCT**T**CGGGCAGCATTCGAACC
PqsRS201Fb*	GGTTCGAATGCTGCCCG**A**AGCGGCTGCCGAGGCTG
rt_rpsL-F	CGGCACTGCGTAAGGTATG
rt_rpsL-R	ACTACGCTGTGCTCTTGCAG
RTpqsEAPFW	GATGACCTGTGCCTGTTGG
RTpqsEAPRv	TGCACCTGGGACGGGTC
PqsAE_PAO1FW	ATCGGATCCACGAAGCCCGTGGTTCTTC
PqsAE_PAO1RV	GCAAAGCTTCGGTAACCCCCCTTTTCCCC

*The nucleotides in bold letters and underlined correspond to the changes made to modify the sequence in the construction of PAO1 pqsR S201F

References

1. Quiroz-Morales SE, García-Reyes S, Ponce-Soto GY, Servín-González L, Soberón-Chávez G Tracking the origins of *Pseudomonas aeruginosa* phylogroups by diversity and evolutionary analysis of important pathogenic marker genes. 2022 Diversity 14: 345.
2. Holloway BW. Genetic Recombination in *Pseudomonas aeruginosa*. J Gen Microbiol. 1955. 13:572–581.
3. Soto-Aceves MP, Cocotl-Yañez M, Servín-González S, Soberón-Chávez G . The Rhl quorum sensing system is at the top of the regulatory hierarchy under phosphate limiting conditions in *Pseudomonas aeruginosa* PAO1. J. Bacteriol. 2021. 203(5): eoo475-20.
4. Diggle SP, Fletcher MP, Cámara M, Williams P. Quorum Sensing, 2011 p. 21–30. *In* Quorum Sensing: Methods and Protocols, Methods in Molecular Biology.
5. Lee DG, Urbach JM, Wu G, Liberati NT, Feinbaum RL, Miyata S, Diggins LT, He J, Saucier M, Deziel E, Friedman L, Li L, Grills G, Montgomery K, Kucherlapati R, Rahme LG, Ausubel FM: Genomic analysis reveals that *Pseudomonas aeruginosa* virulence is combinatorial. Genome Biol 2006, 7: R90.

1. West SEH, Schweizer HP, Dall C, Sample AK, Runyen-Janecky LJ. Construction of improved Escherichia-Pseudomonas shuttle vectors derived from pUC18/19 and sequence of the region required for their replication in Pseudomonas aeruginosa. Gene. 1994. 128:81–86.
2. García-Reyes S, Cocotl-Yañez M, González-Valdez A, Servín-González L, Soberón Chávez G. The PqsR-independent quorum-sensing response of *Pseudomonas aeruginosa* ATCC 9027 outlier-strain reveals new insights on the PqsE effect on RhlR activity. Mol Microbiol. 2021 116(4): 1113-1123. DOI:10.1111/mmi.14797.
